# Genotypic characterization of multiple drug resistant *Escherichia coli* isolates from a pediatric cancer hospital in Egypt

**DOI:** 10.1038/s41598-020-61159-z

**Published:** 2020-03-05

**Authors:** Reem Hassan, Marwa Tantawy, Nouran A. Gouda, Mariam G. Elzayat, Sara Gabra, Amena Nabih, Aya A. Diab, Mohamed El-Hadidi, Usama Bakry, Mohamed R. Shoeb, Mervat Elanany, Lobna Shalaby, Ahmed A. Sayed

**Affiliations:** 1 Molecular Microbiology Unit, Children’s cancer hospital Egypt 57357, Cairo, Egypt; 2 Department of clinical pathology, Faculty of Medicine, Cairo University, Cairo, Egypt; 3 Genomics Program, Children’s cancer hospital Egypt 57357, Cairo, Egypt; 4 Bioinformatics Group, Center of Informatics Sciences (CIS), Nile University, Giza, Egypt; 5 Microbiology Unit, Children’s cancer hospital Egypt 57357, Cairo, Egypt; 6 Infectious disease unit, Children’s cancer hospital Egypt 57357, Cairo, Egypt; 7 Department of pediatric oncology, National cancer Institute, Cairo University, Cairo, Egypt; 8 Department of Biochemistry, Faculty of Science, Ain Shams University, Cairo, Egypt

**Keywords:** Bacterial genetics, Microbial genetics

## Abstract

Infection with multiple drug resistant (MDR) *Escherichia coli* poses a life threat to immunocompromised pediatric cancer patients. Our aim is to genotypically characterize the plasmids harbored in MDR *E. coli* isolates recovered from bacteremic patients of Children’s Cancer Hospital in Egypt 57357 (CCHE 57357). In this study, 21 carbapenem-resistant *E. coli* (CRE) isolates were selected that exhibit Quinolones and Aminoglycosides resistance. Plasmid shot-gun sequencing was performed using Illumina next- generation sequencing platform. Isolates demonstrated resistant to all beta-lactams, carbapenems, aminoglycosides and quinolones. Of the 32 antimicrobial resistant genes identified that exceeded the analysis cutoff coverage, the highest represented genes were *aph(6)-Id, sul2, aph(3″)-Ib, aph(3*′*)-Ia, sul1, dfrA12, TEM-220, NDM-11*. Isolates employed a wide array of resistance mechanisms including antibiotic efflux, antibiotic inactivation, antibiotic target replacements and antibiotic target alteration. Sequenced isolates displayed diverse insertion sequences, including IS26, suggesting dynamic reshuffling of the harbored plasmids. Most isolates carried plasmids originating from other bacterial species suggesting a possible horizontal gene transfer. Only two isolates showed virulence factors with iroA gene cluster which was found in only one of them. Outside the realms of nosocomial infections among patients in hospitals, our results indicate a transfer of resistant genes and plasmids across different organisms.

## Introduction

*Escherichia coli* represents the most frequent sources of blood stream and urinary tract infections worldwide. A continual increase in *E. coli* antibiotic resistance burdens medical facilities throughout the world by causing difficult to treat infections among patients^1,2^. There has been a particular concern regarding the increase in Extended-Spectrum Beta-Lactamase (ESBL)-producing and carbapenem-resistant *E. coli*. carbapenem-resistant *E. coli* (CRE) have become resistant to the majority of available antibiotics, including carbapenems which are a last-resort treatment for multidrug-resistant pathogens. This is often accompanied by resistance to fluoroquinolones and aminoglycosides^3,4^. The increase in antimicrobial resistance (AMR) frequency presents a global healthcare challenge by limiting the choices of antimicrobials that can be used in the treatment of bacterial infections^5,6^.

Mobile elements like transposons, integrons and plasmids frequently carry Multiple Drug Resistance (MDR) genetic motifs. These elements can be transferred from foodborne pathogens to human pathogens, increasing their virulence^7^. This method has enabled the rapid propagation of AMR among several pathogenic bacterial genera to humans, including *E. coli*^8^. CRE-encoding plasmids are now regarded as the primary vector facilitating this transmission between bacteria^9^.

Outlining the entire plasmids along with the genetic structures provides complete sequence information which is crucial for understanding the mechanism of antibiotic resistance and determining the way these elements go through evolutionary changes and horizontal gene transfer to adapt to different host environment^10^.

The emergence of next generation sequencing technology (NGS) has enabled the characterization of bacterial infections as well as identification of virulence factors and antibiotic resistance genes efficiently^11^. NGS has been established as a widespread technique to successfully study the evolutionary relationships of MDR *E. coli* strains from diverse geographical regions. By analyzing the genetic differences in several *E. coli* plasmids recovered from diverse sources, it may be possible to predict resistance phenotypes from the genomic sequences^8,12,13^. Therefore, we describe the use of comparative genomics to track plasmid-mediated antibiotic resistance gene distribution in MDR *E. coli* isolated from bacteremic patients through 2016–2017, at the Children’s Cancer Hospital in Egypt 57357 (CCHE).

## Results and Discussion

**Figure 1:**
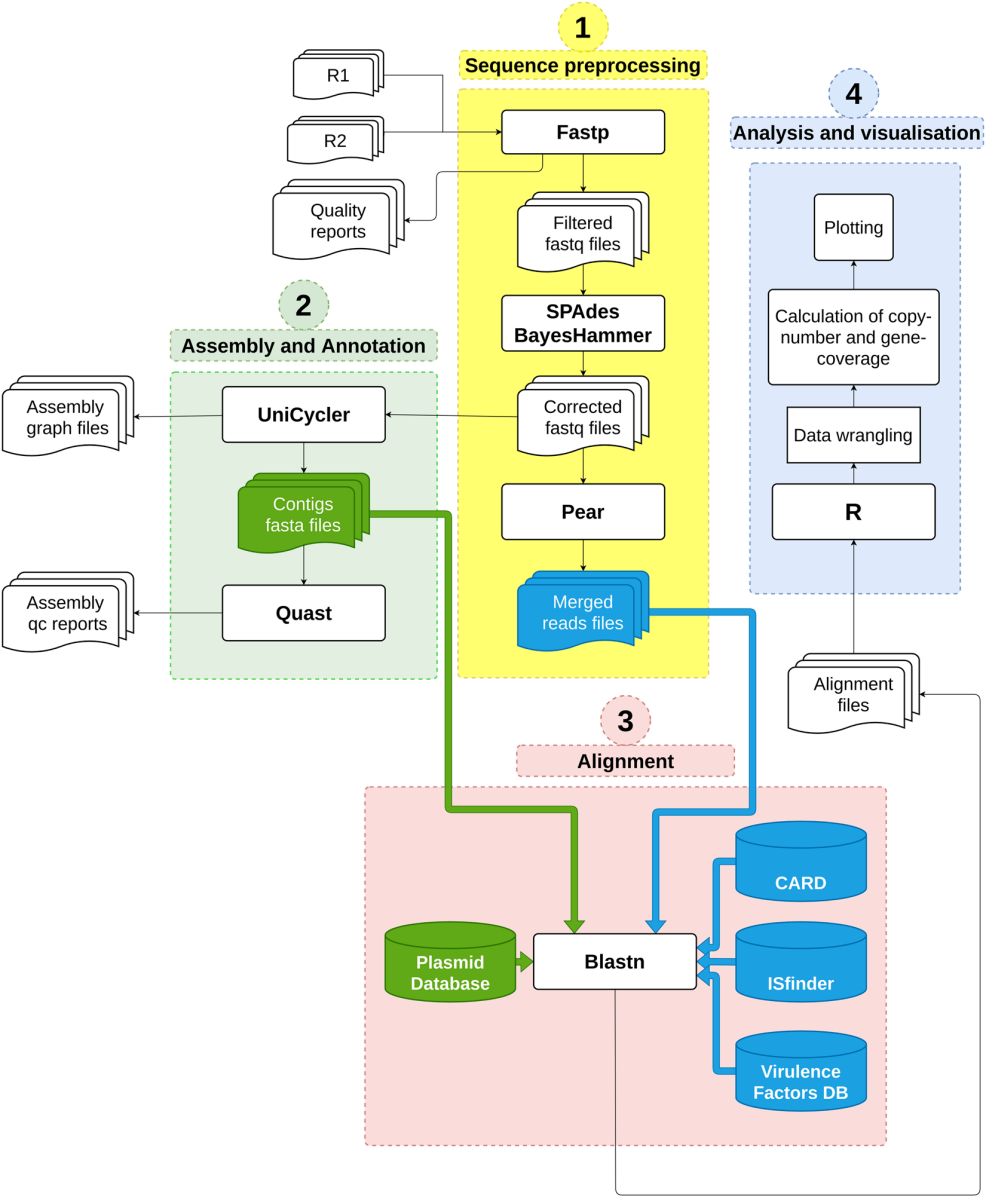
The workflow shows the bioinformatics pipeline used to analyze the shotgun data generated from Illumina MiSeqDx. The pipeline consists of four components: (1) Sequence preprocessing, (2) Assembly and Annotation, (3) Alignment, and (4) Analysis and visualization.

**Figure 2:**
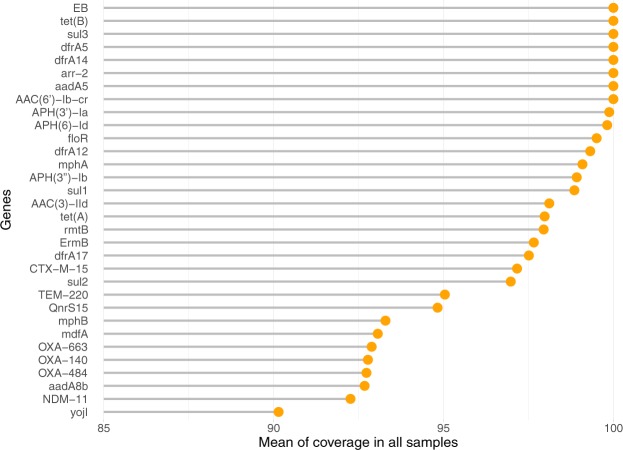
Lollipop plot of the mean coverage for each gene in all samples. Each line represents the mean percent length of a single gene covered by sequencing reads. The mean was calculated from the read coverages in each sample. Genes included in further data analysis exceeded the cutoff coverage of 85%.

After the phenotypic characterization of all 21 isolates by MALDI-TOF MS and AST, we sequenced the plasmids extracted from each isolate in order to correlate the observed resistance with antimicrobial resistance genes. The shotgun sequencing was performed, and the resulting number of reads was 135,315 on an average per sample. The used bioinformatics pipeline to analyze and visualize our data was illustrated in (Fig. 1). We first identified all resistance genes present in each isolate in accordance with 85% minimum coverage (Fig. 2). The majority of isolates expressed AMR genes that were consistent with their observed phenotypic resistance profile.

**Figure 3:**
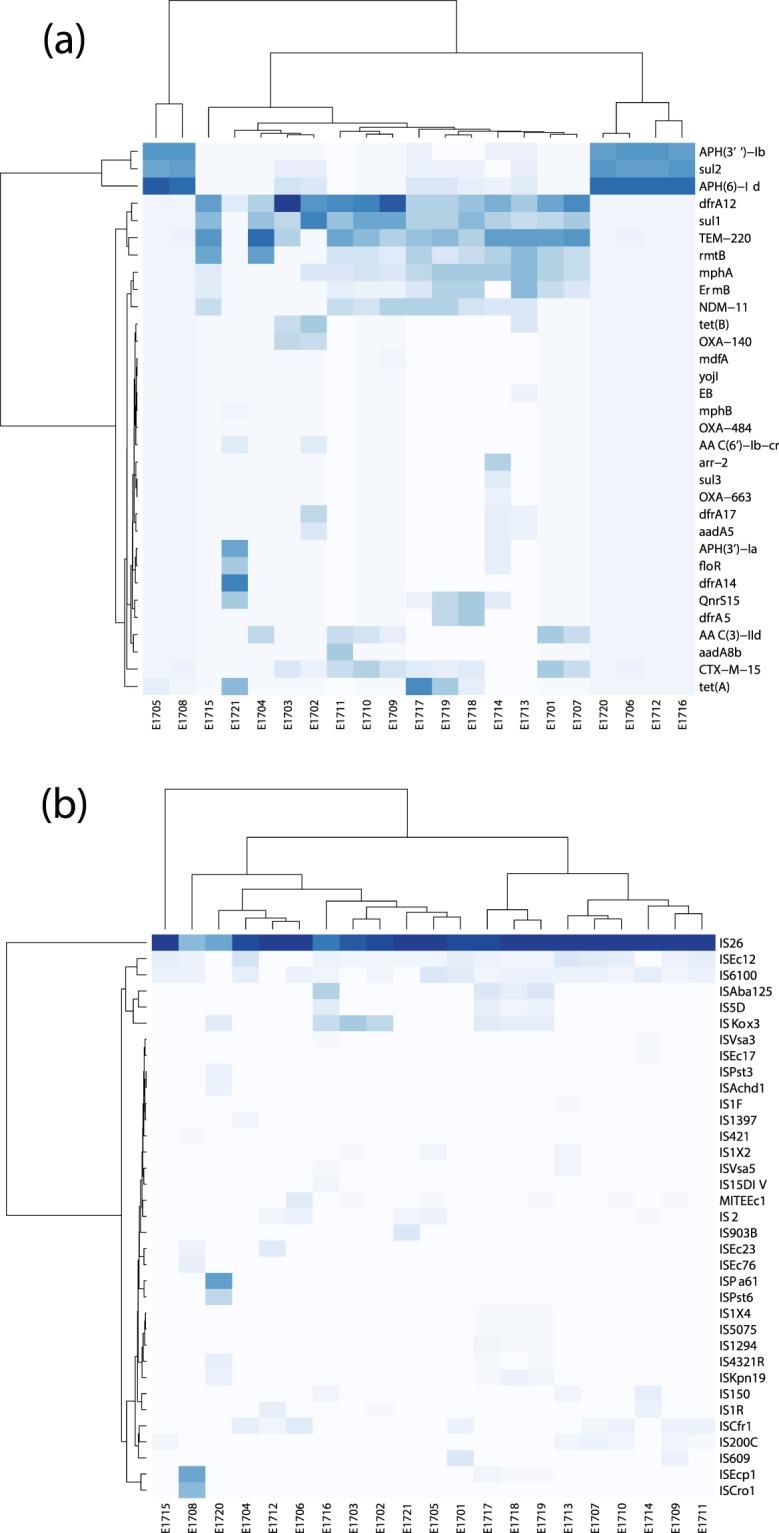
Heatmaps showing (**a)** the relative copy number of each gene and (**b)** each insertion sequence in each sample. Isolates are arranged on the X axis and are given names starting with E17 (*E. coli* from the year 2017), followed by ascending numerical values for each sample. Genes and insertion sequences are plotted on the Y- axis. Genes and insertion sequences with a darker blue colour have a relatively high copy number in a given sample, while genes and insertion sequences with a lighter blue-white colour have a relatively low copy number in comparison to others.

Of the 32 genes that exceeded the cutoff coverage, the highest represented genes were *TEM-220, NDM-11, CTX-M-15* (resistance to carbapenems and beta-lactams), *rmtB, APH(3″)-Ib, APH(6)-Id* (aminoglycoside resistance)*, sul1*, *sul2*, (sulfonamide resistance), *dfrA12, dfrA14*, (diaminopyrimidine antibiotic)*, mphA* (macrolide resistance), *ErmB* (lincosamide, macrolide, streptogramin-b resistance), and *TetA* (tetracycline resistance) (Fig. 3a). While no resistance genes for polymyxins were detected.

We observed a high representation of aminoglycoside resistance gene *aac(6*′*)-Ib-cr* in isolates E1702 and E1721. Both isolates were phenotypically resistant to Tobramycin but isolate E1702 was susceptible to Amikacin and Gentamicin, while isolate E1721 was resistant to both. The *aac(6*′*)-Ib-cr* gene variant is able to induce resistance to aminoglycoside and fluoroquinolone. A gene mutation Leu119Ser is associated with the loss of amikacin resistance while causing resistance to gentamicin. In a previous study, the risk of acquiring *aac(6*′*)-Ib-cr* was significantly associated with gentamicin resistance but also, 12% (2/16) of gentamicin-susceptible isolates harbored *aac(6*′*)-Ib-cr* gene^14^.

Other genes such as *aph(3*′*)Ia* and *aadA5* were highly expressed in these two isolates and they generate resistance to agents of aminoglycoside class such as streptomycin, kanamycin and spectinomycin which are not commonly used in a clinical setting and not tested phenotypically. QnrS14 gene, which confers resistance to quinolones was also found in isolate E1721. Although this gene is more commonly detected in *Salmonella spp*., it was first identified in association with Tn3-like transposon structures on a plasmid in *Shigella flexneri*^15^.

Surprisingly, the isolates clustered into two distinct groups: the first had a high representation of *aph(6)-Id, sul2* and *aph(3″)-Ib*, and included isolates E1705, E1706, E1708, E1712, E1716, E1720. The second had a relatively broad distribution of the remaining highly represented genes: *sul1, dfrA12, TEM-222, NDM-11, rmtB, mphA*, and *Ermb*. Upon examination of the insertion sequences (IS) represented in the 21 isolates, we found that this clustering is lost in the pattern of IS distribution among isolates (Fig. 3b). All isolates carried a high copy number of IS26, followed by ISEc12 (except 1720, 1706, 1714), and IS6100 (except 1703, 1712, 1720, 1721). Meanwhile, ISAba125 and IS5D were highly represented in isolates E1716, E1717, E1718 and E1719. Previous studies showed that most of the resistance genes common among our isolates are frequently flanked by these insertion sequences on different occasions. For example, ISAba125 and ISKox3, in association with bla-NDM^16,17^, IS*ecp1*-*bla*CTX-M-15^18^, IS6100 present within the tnpR gene of the strR transposon Tn5393b, where it was shown to increase the expression of the strA-strB genes^19^.

**Figure 4:**
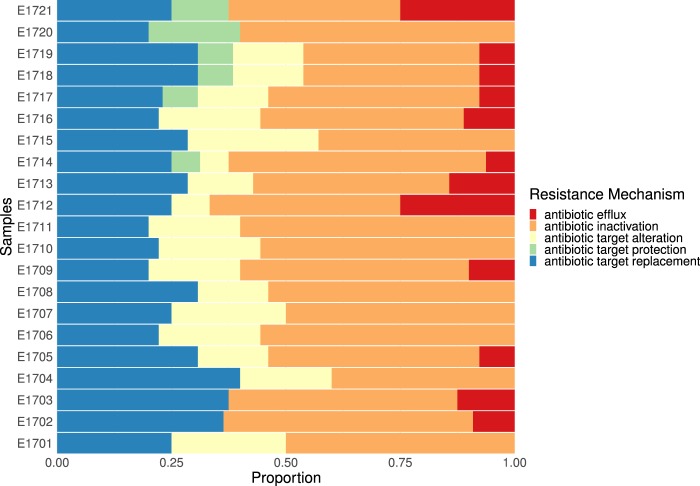
Stacked bar graph showing the resistance mechanisms represented in each isolate after grouping of the AMR genes by mechanism. Each colour represents a mechanism of resistance: blue: antibiotic target replacement, green: antibiotic target protection, yellow: antibiotic target alteration, orange: antibiotic inactivation, red: antibiotic efflux.

IS26 (the most abundant in the studied isolates along with other transposable ISEcp1c and IS6100) plays a major role in the global spread of the class 1 integron as it flanks qacE11, sul1, dfrA and aadA genes^20^. Many studies demonstrated that the presence of IS26 in a resistance site acts to attract resistance genes for integration and encourage shuffling between MDR organisms. This explains why IS26 is often used to trace the evolution of plasmid clones^21,22^. Mapping of insertion sequences exact position with relation to the identified AMR genes requires deeper sequencing coverage. Furthermore, we were able to use the dataset describing AMR gene copy number to determine the main mechanisms of antibiotic resistance employed by each *E. coli* isolate (Fig. 4). These were found to be antibiotic efflux, antibiotic inactivation, antibiotic target replacements, antibiotic target protection and antibiotic target alteration. While all isolates carry genes that are responsible for antibiotic inactivation (most represented mechanism), antibiotic target replacements and antibiotic target alteration, and some carry genes that affect antibiotic efflux (all except E1701, E1704, E1706, E1707, E1708, E1710, E1711, E1715, E1720), few carry genes that affect antibiotic target protection (E1714, E1717, E1718, E1719, E1720, E1721). Interestingly, the six isolates clustering together (E1705, E1706, E1708, E1712, E1716, E1720), did not show the same AMR mechanism pattern. This was expected since those isolates, despite carrying predominantly the same three genes, also carry varying copy numbers of the remaining 32 genes represented in this dataset which may confer varying mechanisms of resistance in these isolates.

Using Virulence Finder we identified the virulence factors found in each isolate (Table 1). GspJ virulence gene was found in isolate E1713 only and it encodes Type II secretion system protein GspJ which is a component of an operon of genes GspC-O. They are not normally expressed but are homologous to those in Klebsiella oxytoca and Aeromonase hydrophila^23^. GspJ is one of the minor pseudopilins forming the pseudopilus in many of the Gram-negative bacteria. GspI and GspK pseudopilins form a complex which plays an important role in assembly of pseudopilus that is thought to drive the secretion process^24^.

**Table 1:** Virulence Factors.

	E	E	E	E	E	E	E	E	E	E	E	E	E	E	E	E	E	E	E	E	E
	1	1	1	1	1	1	1	1	1	1	1	1	1	1	1	1	1	1	1	1	1
	7	7	7	7	7	7	7	7	7	7	7	7	7	7	7	7	7	7	7	7	7
	0	0	0	0	0	0	0	0	0	1	1	1	1	1	1	1	1	1	1	2	2
	1	2	3	4	5	6	7	8	9	0	1	2	3	4	5	6	7	8	9	0	1
gspJ													•								
iroB														•							
iroC														•							
iroD														•							
iroE														•							
iroN														•							
iucB														•							
iucC														•							
iucD														•							
iutA														•							

Isolate E1714 contained five virulence genes iroB, iroC, iroD, iroE, and iroN, commonly present in *E. coli*, involved in iron ion binding and signaling receptor activity. They are commonly present on IncFI and ColV2-K94 plasmids^25^. They act through formation of iroA gene cluster that permits the pathogen to acquire iron from the host avoiding the innate immune system proteins such as NGAL (also known as lipocalin 2) that sequester the bacterial iron siderophores. The iroA cluster modifies the enterobactin before its secretion through subsequent processes of glucosylation and linearization performed using the 5 virulence genes^26^.

**Figure 5:**
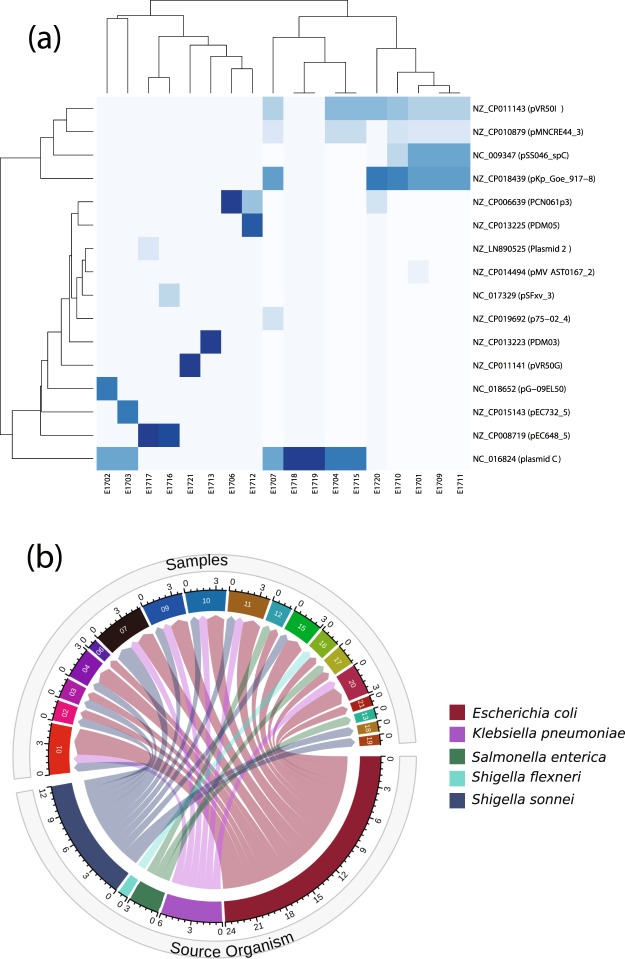
(**a**) Heatmap showing the relative copy number of each plasmid in each sample. Isolates are arranged on the X axis, plasmids are plotted on the Y- axis. Plasmids with a darker blue colour have a relatively high copy number in a given sample, while plasmids with a lighter blue-white colour have a relatively low copy number in comparison to others. (**b)** Graph showing the source organism of each plasmid identified in each isolate. Isolates are arranged by identifier in the top half, source organisms are arranged in the bottom half.

When examining plasmids, pVR50I (*E. coli*), pMNCRE44_3 (*E. coli*), pSS046_spC (*Shigella sonnei*), pKp_Goe_917-8 (*Klebsiella pneumoniae*), were found abundantly in isolates E1701, E1709, E1710, E1720, while Plasmid C (*Shigella sonnei*) was abundant in isolates; E1702, E1703, E1704, E1707, E1715, E1718, E1719 (Fig. 5a). In addition, pCN061p3 (6,222 bp size in *E. coli*) was highly represented in isolates E1712, E1716, E1720 which clustered together and harbored the resistance genes sul and strAB^27^. These plasmids were found in previous studies carrying variable resistance genes^28–30^ that is consistent with the phenotypic and genetic characterization of AMR in these isolates.

Horizontal gene transfer (HGT) enables microbial species to gain new genetic material other than their clone^31,32^. This leads to bacterial genome diversity and increases bacterial fitness. HGT can lead to the transfer of virulence genes as well as antimicrobial resistance genes increasing bacterial pathogenic traits^33^. Few studies encroach HGT in clinical settings and how it affects (mainly through plasmids) the burden of antimicrobial resistance in many problematic pathogens.

In the interest of understanding the extent to which plasmids represented in the 21 isolates originated from *E. coli*, we used the data generated using PlasmidFinder to track sources of each plasmid in each isolate, using their origins of replication. Since all isolates belong to the species *E. coli*, the majority of plasmids were of *E. coli* origin (Fig. 5b). However, all isolates carry plasmids from other bacterial origins, such as *Klebsiella pneumoniae*, *Shigella sonnei*, *Shigella flexneri* and *Salmonella enterica*. These are among the most common human pathogenic species.

Although this may be largely due to HGT, commonly occurring in nature, it may also be attributed to the fact that *E. coli* residing in human gut (that later causes infection) are present in combination with other Gram-positive and Gram-negative organisms^34^ with possible plasmids transfer through conjugation (direct cell contact)^35^.

A preceding blood stream infection with another organism was recorded in 3 other cases as well; E1701 (preceding *Enterococcus faecium* by 6 days, *Pseudomonas spp*. by 9 days), E1708 (preceding *Salmonella spp*. by 5 days) and E1720 (preceding *Acinetobacter baumannii* by 4 days), for which all patients received antimicrobial therapy or prophylactic therapy due to immunosuppression condition. Some studies incriminate combined antibiotics use as an inducer for conjugation between Gram-negative species^36,37^. Antibiotics may also be involved in cell signaling involved in the transcription of virulence genes, DNA repair, and DNA transfer^36,38,39^. Fluoroquinolones that cause DNA damage can stimulate the SOS DNA repair response. SOS genes were postulated to stimulate conjugation as well^40^.

In an attempt to further characterize the two groups of isolates, we examined the sample source for each isolate. Despite the genetic similarity between bacterial isolates in the present study, no epidemiological link in terms of time and place could be postulated. It has been previously hypothesized that possible circulation of special strains can occur in hospitals rather than nosocomial transmission between patients when no epidemiological relation can be demonstrated between genetically similar isolates^41^. However, we did observe that the six isolates clustering together were from patients who had no previous infection. This may explain the lack of diversity in the distribution of AMR genes in these isolates since the infecting population of bacteria had not been previously exposed to vigorous antibiotics and did not need to evolve a wide array of AMR genes.

Despite the genetic similarity between bacterial isolates in the present study, no epidemiological link in terms of time and place could be postulated. This was previously hypothesized that possible circulation of special strains can occur in hospitals rather than nosocomial transmission^41^. *E. coli* is an important nosocomial pathogen with extended antimicrobial resistance profile, using molecular typing methods can link these MDR isolates with specific clinical features, based on phylogenetic relatedness to point out outbreak source and dissemination pattern. We recommend the presence of an online source for bacterial typing that offers a rapid source tracing tool using plasmid profile as other tools using genomic data^42^.

## Methods

### Bacterial isolation & identification

Each patient admitted to the Children’s Cancer Hospital Egypt 57357 (CCHE57357) with suspected blood stream infection (BSI) undergoes blood culture testing using BACTEC blood culture system 9240 (Becton Dickinson Diagnostic Instrument Systems, Sparks, Md). All procedures performed in the study involving human participants were in accordance with the ethical standards of the institutional research committee and with the 1964 Declaration of Helsinki and its later amendments or comparable ethical standards. Each patient’s guardian signed informed consent as an assent to participate in the current study, all of which were approved by the Institutional Review Board at CCHE57357. Positive Blood culture bottles were subjected to subculture on solid media (OxoidTM) according to standards for bacterial isolation^43^. Bacterial species identification was performed in duplicate, with tests performed simultaneously on the same target slide using the MALDI-TOF Vitek MS (bioMérieux) and analyzed on the Vitek MS IVD system (bioMérieux; Marcy l′Etoile, France) according to manufacturer’s instructions. All experimental protocols carried out in this study were approved by CCHE 57357 Scientific and Medical Advisory Committee (SMAC).

### Antimicrobial susceptibility testing (AST)

Testing of *E. coli* isolates was performed using Vitek 2 AST cards GN73 (bioMérieux SA, Marcy l′Étoile, France) according to the manufacturer’s instructions. Interpretation of results was done according to CLSI guidelines^44,45^. Isolates were defined as CRE pattern when showing resistance to Carbapenem, Penicillins and Cephalosporins. We selected 21 CRE isolates that have shown Quinolones and Aminoglycosides resistance (MDR)^3^. All isolates were 100% susceptible to polymixins. These were stored in 15% glycerol broth for later molecular testing.

### Plasmid extraction

A single colony from a freshly streaked plate was inoculated in 2 ml of Luria-Bertani (LB) medium to prepare the bacterial culture. After incubation overnight at 37 °C with shaking, the culture was subjected to plasmid DNA isolation. The bacterial pellet was harvested by centrifugation at 5,000 × g for 10 minutes at 4 °C then resuspended using 2 mL resuspension buffer. The plasmid DNA was extracted and purified using The GeneJET Plasmid MidiPrep Kit (Thermo Scientific, USA) according to the manufacturer’s instructions and stored at −20 °C until used for library preparation. The plasmid visualization was performed using 1% agarose gel to confirm the existence of plasmid.

### Library preparation and next generation sequencing

The extracted plasmid DNA was prepared for sequencing using the Nextera XT DNA library preparation kit (Illumina, USA). The Nextera XT DNA library preparation kit uses transposome to fragment and tag the input DNA and adding a unique adapter. The fragmented DNA was amplified by 12 cycles of PCR reaction which adds the primers and index sequences for dual-indexed sequencing of pooled libraries. Samples were normalized and pooled, then sequenced using the Illumina MiSeq, using 150-base paired-end reads. The sample preparation and sequencing were performed according to the manufacturer’s protocol.

### Computational analysis

#### Sequence pre-processing and quality control

The quality control of the generated read-pairs for each sample were performed using Fastp (v0.20.0) and high quality (mean per-base-sequence-quality > = 30) filtered read-pairs were then corrected using BayesHammer (SPAdes v3.13.0) (Supplementary Table 1)^46,47^. After that, the corrected read-pairs were merged using PEAR (v0.9.6) into a single read to facilitate the alignment process^48^. Finally, all the reads were concatenated together into a single file, after designated names to read headers (Fig. 1).

#### Assembly and annotation

The read-pairs were assembled using UniCycler (v0.4.8) using default parameters with adjusted Kmer count to twenty^49^. The UniCycler’s output files (contig fasta files) were used in plasmid alignment and characterization. The assessment of the assembled files was carried out by QUAST (v5.0.2) (Supplementary Table 2)^50^.

#### Alignment

The profiling of AMR genes took place by aligning the reads using Blast (v2.9.0) against CARD database (v3.0.7) (Supplementary Table 3)^51,52^. Sequenced plasmids were characterized by aligning the contigs of each sample against the database of plasmid sequences (v2) (Supplementary Table 4)^53^. In addition, plasmid-borne virulence factors and insertion sequences were detected by aligning the reads to the Virulence Factors Database (update 01/24/2020) and ISFinder database (update 09/25/2019), respectively (Supplementary Table 5)^54,55^. All alignment results were saved in tabular form.

#### Analysis and visualization

The results of the alignments were imported to Rstudio (version 1.2.1335) for further analysis (Fig. 1)^56^. Reads with less than 90% identity or 1e^−4^ e-value were filtered-out. The gene-coverage was defined as the percentage of covered bases in each gene. Then, gene-copy-number was calculated by dividing the number of reads aligned to each gene by its length. As a validation of our computational methods, we calculated the mean coverage of each gene in all 21 isolates. We set a cutoff coverage of 85%. Hence, only genes with sequencing reads covering over 85% of the length of the gene were included in any downstream analyses (Fig. 2). In plasmids downstream analysis, the source organism for each aligned plasmid in each sample was retrieved from NCBI Nucleotide database by using the plasmid accession number and used to generate the chord graph using circlize package (v0.4.8) (Fig. 5b)^57^. The other results of the analysis are visualized in heatmaps, stacked bar plots and lollipop charts using ggplot2 package (v3.2.1)^58^ (Figs. 3–5).

## Conclusion

Plasmid shot-gun sequencing of MDR *E. coli* isolates from CCHE57357 revealed that the most predominant antimicrobial resistant mechanism was antibiotic inactivation, followed by antibiotic target replacements and antibiotic target alteration, then antibiotic efflux, but only a few carry genes that induce antibiotics target protection. In addition to the threat of nosocomial infection that involves the transfer of strains between different patients in hospitals, our results indicate a transfer of resistant genes and plasmids across different organisms. In the future, changed plasmid resistance profile can be used as an adjunctive indicator to differentiate between a prolonged BSI episode and a new one with the same organism.

## Supplementary information

Dataset 1.

## Data Availability

All data generated, analyzed during this study are included in this article and published online on NCBI with SRA Accession Number: PRJNA603448.
