# Author Correction: Genotypic characterization of multiple drug resistant *Escherichia coli* isolates from a pediatric cancer hospital in Egypt

**DOI:** 10.1038/s41598-020-64153-7

**Published:** 2020-04-29

**Authors:** Reem Hassan, Marwa Tantawy, Nouran A. Gouda, Mariam G. Elzayat, Sara Gabra, Amena Nabih, Aya A. Diab, Mohamed El-Hadidi, Usama Bakry, Mohamed R. Shoeb, Mervat Elanany, Lobna Shalaby, Ahmed A. Sayed

**Affiliations:** 1 Molecular Microbiology Unit, Children’s cancer hospital Egypt 57357, Cairo, Egypt; 2 Department of clinical pathology, Faculty of Medicine, Cairo University, Cairo, Egypt; 3 Genomics Program, Children’s cancer hospital Egypt 57357, Cairo, Egypt; 4 Bioinformatics Group, Center of Informatics Sciences (CIS), Nile University, Giza, Egypt; 5 Microbiology Unit, Children’s cancer hospital Egypt 57357, Cairo, Egypt; 6 Infectious disease unit, Children’s cancer hospital Egypt 57357, Cairo, Egypt; 7 Department of pediatric oncology, National cancer Institute, Cairo University, Cairo, Egypt; 8 Department of Biochemistry, Faculty of Science, Ain Shams University, Cairo, Egypt

Correction to: *Scientific Reports* 10.1038/s41598-020-61159-z, published online 05 March 2020

The original version of this Article contained an error in Affiliation 4, which was incorrectly given as ‘Nile University, Giza, Egypt’. The correct affiliation is listed below:

Bioinformatics Group, Center of Informatics Sciences (CIS), Nile University, Giza, Egypt

This error has now been corrected in the HTML and PDF versions of the Article.

