# Supplementary material for: Genotypic characterization of multiple drug resistant Escherichia coli isolates from a pediatric cancer hospital in Egypt
Source: Sci Rep. 2020 Mar 5;10:4165. doi: 10.1038/s41598-020-61159-z (PMC7057982; doi:10.1038/s41598-020-61159-z)
Supplement: Supplementary file 1 — Dataset 1. [file 41598_2020_61159_MOESM1_ESM.pdf]

# Genotypic characterization of multiple drug resistant *Escherichia coli* isolates from a pediatric cancer hospital in Egypt

**Authors: Reem Hassan, Marwa Tantawy, Nouran A. Gouda, Mariam G. Elzayat, Sara Gabra, Amena Nabih, Aya A. Diab, Mohamed El-Hadidi, Usama Bakry, Mohamed R. Shoeb, Mervat El-Annany, Lobna Shalaby, Ahmed A. Sayed.**

**Supplementary Table 1: The table shows the quality of all samples before and after filtration and filtering result using Fastp**

| Samples | E1701 | E1702 | E1703 | E1704 | E1705 | E1706 | E1707 | E1708 | E1709 | E1710 | E1711 |
|---|---|---|---|---|---|---|---|---|---|---|---|
| **General** | | | | | | | | | | | |
| sequencing: | paired end (151 cycles + 151 cycles) | paired end (151 cycles + 151 cycles) | paired end (151 cycles + 151 cycles) | paired end (151 cycles + 151 cycles) | paired end (151 cycles + 151 cycles) | paired end (151 cycles + 151 cycles) | paired end (151 cycles + 151 cycles) | paired end (151 cycles + 151 cycles) | paired end (151 cycles + 151 cycles) | paired end (151 cycles + 151 cycles) | paired end (151 cycles + 151 cycles) |
| mean length before filtering: | 146bp, 146bp | 144bp, 144bp | 145bp, 145bp | 147bp, 147bp | 145bp, 145bp | 143bp, 143bp | 144bp, 144bp | 143bp, 143bp | 145bp, 145bp | 144bp, 144bp | 145bp, 145bp |
| mean length after filtering: | 146bp, 146bp | 144bp, 144bp | 145bp, 145bp | 147bp, 147bp | 145bp, 145bp | 143bp, 143bp | 144bp, 144bp | 143bp, 143bp | 145bp, 145bp | 144bp, 144bp | 145bp, 145bp |
| duplication rate: | 0.728665% | 0.755800% | 0.790925% | 0.497097% | 1.084178% | 0.442796% | 0.635441% | 0.909171% | 0.617148% | 0.608885% | 0.554971% |
| Insert size peak: | 219 | 201 | 226 | 240 | 216 | 187 | 215 | 239 | 204 | 191 | 196 |
| **Before filtering** | | | | | | | | | | | |
| total reads: | 147.506000 K | 92.860000 K | 108.068000 K | 92.672000 K | 112.372000 K | 108.344000 K | 171.046000 K | 160.498000 K | 176.438000 K | 189.514000 K | 166.554000 K |
| total bases: | 21.555013 M | 13.392556 M | 15.773915 M | 13.706835 M | 16.313564 M | 15.575082 M | 24.785905 M | 22.954163 M | 25.664897 M | 27.373007 M | 24.255634 M |
| Q20 bases: | 20.693177 M (96.001691%) | 12.810835 M (95.656386%) | 15.177961 M (96.221902%) | 13.154419 M (95.969777%) | 15.668486 M (96.045757%) | 15.041320 M (96.572975%) | 23.704390 M (95.636572%) | 21.993765 M (95.816018%) | 24.180725 M (94.217113%) | 25.782392 M (94.189111%) | 22.703201 M (93.599701%) |
| Q30 bases: | 20.434483 M (94.801534%) | 12.655956 M (94.499930%) | 15.007970 M (95.144230%) | 12.987820 M (94.754332%) | 15.492179 M (94.965018%) | 14.880152 M (95.538194%) | 23.393432 M (94.381997%) | 21.703480 M (94.551389%) | 23.755220 M (92.559187%) | 25.327294 M (92.526532%) | 22.274134 M (91.830764%) |
| GC content: | 49.693777% | 45.533384% | 45.846196% | 49.609527% | 47.041094% | 49.611989% | 49.432373% | 48.828498% | 49.891687% | 49.799439% | 49.396058% |
| **After filtering** | | | | | | | | | | | |
| total reads: | 147.182000 K | 92.604000 K | 107.724000 K | 92.364000 K | 112.128000 K | 108.066000 K | 170.446000 K | 160.086000 K | 175.714000 K | 188.750000 K | 165.796000 K |
| total bases: | 21.498516 M | 13.348305 M | 15.716356 M | 13.656585 M | 16.274046 M | 15.536905 M | 24.693604 M | 22.897110 M | 25.546646 M | 27.247088 M | 24.132938 M |
| Q20 bases: | 20.655390 M (96.078213%) | 12.782672 M (95.762511%) | 15.140601 M (96.336587%) | 13.120823 M (96.076896%) | 15.643820 M (96.127417%) | 15.017586 M (96.657513%) | 23.645314 M (95.754812%) | 21.960534 M (95.909632%) | 24.103278 M (94.350068%) | 25.700760 M (94.324795%) | 22.624744 M (93.750475%) |
| Q30 bases: | 20.401714 M (94.898243%) | 12.631117 M (94.627123%) | 14.974864 M (95.282036%) | 12.958680 M (94.889608%) | 15.470582 M (95.062912%) | 14.859280 M (95.638610%) | 23.342029 M (94.526619%) | 21.675191 M (94.663436%) | 23.688712 M (92.727288%) | 25.257358 M (92.697458%) | 22.207246 M (92.020483%) |
| GC content: | 49.691588% | 45.528163% | 45.831941% | 49.602642% | 47.028975% | 49.615995% | 49.425778% | 48.830324% | 49.876222% | 49.783162% | 49.370578% |
| **Filtering result** | | | | | | | | | | | |
| reads passed filters: | 147.182000 K (99.780348%) | 92.604000 K (99.724316%) | 107.724000 K (99.681682%) | 92.364000 K (99.667645%) | 112.128000 K (99.782864%) | 108.066000 K (99.743410%) | 170.446000 K (99.649217%) | 160.086000 K (99.743299%) | 175.714000 K (99.589658%) | 188.750000 K (99.596864%) | 165.796000 K (99.544892%) |
| reads with low quality: | 322 (0.218296%) | 248 (0.267069%) | 330 (0.305363%) | 302 (0.325881%) | 234 (0.208237%) | 264 (0.243668%) | 580 (0.339090%) | 402 (0.250470%) | 710 (0.402408%) | 744 (0.392583%) | 746 (0.447903%) |
| reads with too many N: | 0 (0.000000%) | 0 (0.000000%) | 0 (0.000000%) | 0 (0.000000%) | 0 (0.000000%) | 0 (0.000000%) | 0 (0.000000%) | 0 (0.000000%) | 0 (0.000000%) | 0 (0.000000%) | 0 (0.000000%) |
| reads too short: | 2 (0.001356%) | 8 (0.008615%) | 14 (0.012955%) | 6 (0.006474%) | 10 (0.008899%) | 14 (0.012922%) | 20 (0.011693%) | 10 (0.006231%) | 14 (0.007935%) | 20 (0.010553%) | 12 (0.007205%) |

| Samples | E1712 | E1713 | E1714 | E1715 | E1716 | E1717 | E1718 | E1719 | E1720 | E1721 |
|---|---|---|---|---|---|---|---|---|---|---|
| **General** | | | | | | | | | | |
| sequencing: | paired end (151 cycles + 151 cycles) | paired end (151 cycles + 151 cycles) | paired end (151 cycles + 151 cycles) | paired end (151 cycles + 151 cycles) | paired end (151 cycles + 151 cycles) | paired end (151 cycles + 151 cycles) | paired end (151 cycles + 151 cycles) | paired end (151 cycles + 151 cycles) | paired end (151 cycles + 151 cycles) | paired end (151 cycles + 151 cycles) |
| mean length before filtering: | 146bp, 146bp | 147bp, 147bp | 144bp, 144bp | 142bp, 142bp | 146bp, 146bp | 145bp, 145bp | 146bp, 146bp | 144bp, 144bp | 146bp, 146bp | 145bp, 145bp |
| mean length after filtering: | 146bp, 146bp | 147bp, 147bp | 144bp, 144bp | 142bp, 142bp | 146bp, 146bp | 145bp, 145bp | 146bp, 146bp | 144bp, 144bp | 146bp, 146bp | 145bp, 145bp |
| duplication rate: | 0.321816% | 0.257624% | 1.140126% | 0.873080% | 0.601054% | 0.428524% | 0.522179% | 0.418534% | 0.063826% | 0.434797% |
| Insert size peak: | 251 | 226 | 207 | 196 | 222 | 250 | 203 | 239 | 207 | 220 |
| **Before filtering** | | | | | | | | | | |
| total reads: | 103.976000 K | 89.322000 K | 160.632000 K | 215.450000 K | 118.992000 K | 166.342000 K | 128.944000 K | 143.684000 K | 83.914000 K | 104.494000 K |
| total bases: | 15.212257 M | 13.187536 M | 23.256105 M | 30.623275 M | 17.428649 M | 24.234866 M | 18.948536 M | 20.772085 M | 12.313493 M | 15.169174 M |
| Q20 bases: | 14.709235 M (96.693311%) | 12.704665 M (96.338429%) | 22.359766 M (96.145791%) | 28.791353 M (94.017877%) | 16.302830 M (93.540412%) | 22.551202 M (93.052720%) | 17.739321 M (93.618425%) | 18.895978 M (90.968133%) | 11.776208 M (95.636616%) | 14.301071 M (94.277190%) |
| Q30 bases: | 14.530632 M (95.519238%) | 12.544711 M (95.125511%) | 22.091592 M (94.992657%) | 28.301837 M (92.419367%) | 16.005294 M (91.833245%) | 22.124302 M (91.291208%) | 17.421559 M (91.941451%) | 18.446477 M (88.804167%) | 11.571737 M (93.976072%) | 14.062976 M (92.707592%) |
| GC content: | 52.578299% | 50.648984% | 46.230519% | 49.350310% | 49.065346% | 49.553334% | 49.848627% | 49.415429% | 59.035068% | 49.546917% |
| **After filtering** | | | | | | | | | | |
| total reads: | 103.482000 K | 88.906000 K | 160.250000 K | 214.570000 K | 118.442000 K | 165.426000 K | 128.320000 K | 142.554000 K | 83.162000 K | 104.000000 K |
| total bases: | 15.136040 M | 13.122638 M | 23.195988 M | 30.484575 M | 17.347317 M | 24.097860 M | 18.849793 M | 20.605437 M | 12.201033 M | 15.098580 M |
| Q20 bases: | 14.658489 M (96.844941%) | 12.662008 M (96.489806%) | 22.320731 M (96.226688%) | 28.707389 M (94.170212%) | 16.253266 M (93.693255%) | 22.467432 M (93.234138%) | 17.677593 M (93.781364%) | 18.797907 M (91.227898%) | 11.705030 M (95.934746%) | 14.256847 M (94.425085%) |
| Q30 bases: | 14.486740 M (95.710239%) | 12.507633 M (95.313404%) | 22.057279 M (95.090923%) | 28.230529 M (92.605946%) | 15.963248 M (92.021423%) | 22.053306 M (91.515620%) | 17.368873 M (92.143574%) | 18.364313 M (89.123628%) | 11.511779 M (94.350855%) | 14.025190 M (92.890788%) |
| GC content: | 52.549075% | 50.617452% | 46.217432% | 49.332444% | 49.036488% | 49.514098% | 49.807120% | 49.358347% | 58.972794% | 49.530307% |
| **Filtering result** | | | | | | | | | | |
| reads passed filters: | 103.482000 K (99.524890%) | 88.906000 K (99.534269%) | 160.250000 K (99.762189%) | 214.570000 K (99.591553%) | 118.442000 K (99.537784%) | 165.426000 K (99.449327%) | 128.320000 K (99.516069%) | 142.554000 K (99.213552%) | 83.162000 K (99.103844%) | 104.000000 K (99.527246%) |
| reads with low quality: | 494 (0.475110%) | 408 (0.456774%) | 376 (0.234075%) | 856 (0.397308%) | 550 (0.462216%) | 908 (0.545863%) | 620 (0.480829%) | 1.122000 K (0.780880%) | 742 (0.884239%) | 490 (0.468926%) |
| reads with too many N: | 0 (0.000000%) | 0 (0.000000%) | 0 (0.000000%) | 0 (0.000000%) | 0 (0.000000%) | 0 (0.000000%) | 0 (0.000000%) | 0 (0.000000%) | 0 (0.000000%) | 0 (0.000000%) |
| reads too short: | 0 (0.000000%) | 8 (0.008956%) | 6 (0.003735%) | 24 (0.011139%) | 0 (0.000000%) | 8 (0.004809%) | 4 (0.003102%) | 8 (0.005568%) | 10 (0.011917%) | 4 (0.003828%) |

**Supplementary Table 2: The table shows the evaluation of all assemblies using QUAST**

| Samples | E1701 | E1702 | E1703 | E1704 | E1705 | E1706 | E1707 | E1708 | E1709 | E1710 | E1711 | E1712 | E1713 | E1714 | E1715 | E1716 | E1717 | E1718 | E1719 | E1720 | E1721 |
|---|---|---|---|---|---|---|---|---|---|---|---|---|---|---|---|---|---|---|---|---|---|
| # contigs (>= 0 bp) | 39 | 42 | 33 | 63 | 46 | 74 | 38 | 26 | 36 | 35 | 35 | 111 | 45 | 39 | 69 | 27 | 22 | 51 | 59 | 203 | 29 |
| # contigs (>= 1000 bp) | 36 | 38 | 32 | 58 | 36 | 61 | 32 | 19 | 29 | 31 | 26 | 110 | 27 | 37 | 31 | 24 | 20 | 35 | 43 | 191 | 28 |
| # contigs (>= 5000 bp) | 15 | 12 | 9 | 10 | 11 | 11 | 18 | 7 | 11 | 15 | 12 | 10 | 8 | 11 | 7 | 14 | 11 | 15 | 19 | 6 | 9 |
| # contigs (>= 10000 bp) | 8 | 6 | 6 | 6 | 5 | 4 | 9 | 3 | 7 | 7 | 9 | 2 | 3 | 4 | 1 | 10 | 7 | 10 | 11 | 2 | 3 |
| # contigs (>= 25000 bp) | 3 | 2 | 3 | 3 | 0 | 2 | 4 | 2 | 4 | 4 | 4 | 0 | 3 | 1 | 0 | 4 | 2 | 4 | 4 | 1 | 3 |
| # contigs (>= 50000 bp) | 2 | 0 | 0 | 1 | 0 | 0 | 1 | 1 | 3 | 2 | 3 | 0 | 1 | 0 | 0 | 0 | 1 | 2 | 0 | 0 | 0 |
| Total length (>= 0 bp) | 341335 | 223872 | 211319 | 302986 | 165942 | 236491 | 344471 | 186960 | 342323 | 340883 | 345579 | 269499 | 201392 | 195421 | 126543 | 268923 | 306846 | 370173 | 366139 | 325790 | 201654 |
| Total length (>= 1000 bp) | 339846 | 221850 | 210562 | 300650 | 160295 | 230736 | 340495 | 183526 | 338105 | 337865 | 339922 | 268678 | 190787 | 193961 | 106366 | 267981 | 305564 | 359979 | 354402 | 318707 | 200913 |
| Total length (>= 5000 bp) | 289467 | 166676 | 170914 | 203447 | 98265 | 130858 | 301921 | 151270 | 291652 | 302337 | 308185 | 85421 | 145830 | 123910 | 52231 | 247482 | 280720 | 308723 | 305279 | 68243 | 146375 |
| Total length (>= 10000 bp) | 240855 | 123711 | 150444 | 176181 | 58825 | 87552 | 240413 | 125539 | 262499 | 242943 | 287630 | 26831 | 111352 | 77117 | 10108 | 216424 | 254867 | 279250 | 249985 | 41940 | 104990 |
| Total length (>= 25000 bp) | 167846 | 60427 | 107123 | 130362 | 0 | 60899 | 168989 | 105486 | 211401 | 192797 | 216006 | 0 | 111352 | 33755 | 0 | 126016 | 165095 | 180099 | 135584 | 29438 | 104990 |
| Total length (>= 50000 bp) | 135935 | 0 | 0 | 55505 | 0 | 0 | 57720 | 64025 | 183808 | 126127 | 169247 | 0 | 50019 | 0 | 0 | 0 | 129041 | 106380 | 0 | 0 | 0 |
| # contigs | 39 | 42 | 33 | 63 | 46 | 74 | 38 | 26 | 36 | 35 | 35 | 111 | 45 | 39 | 69 | 27 | 22 | 51 | 59 | 203 | 29 |
| Largest contig | 79699 | 30229 | 37335 | 55505 | 12924 | 34498 | 57720 | 64025 | 64300 | 68907 | 59125 | 14093 | 50019 | 33755 | 10108 | 36054 | 129041 | 53740 | 37675 | 29438 | 39474 |
| Total length | 341335 | 223872 | 211319 | 302986 | 165942 | 236491 | 344471 | 186960 | 342323 | 340883 | 345579 | 269499 | 201392 | 195421 | 126543 | 268923 | 306846 | 370173 | 366139 | 325790 | 201654 |
| GC (%) | 48.92 | 48.18 | 47.92 | 48.74 | 51.71 | 49.66 | 48.96 | 49.41 | 48.86 | 48.94 | 48.9 | 51.26 | 49.16 | 51.82 | 51.95 | 48.95 | 50.09 | 50.76 | 50.69 | 58.44 | 50.11 |
| N50 | 17748 | 14678 | 33933 | 15145 | 6116 | 5956 | 23084 | 41461 | 55654 | 28045 | 46759 | 2769 | 29711 | 6741 | 3642 | 19155 | 36054 | 20101 | 18604 | 1409 | 28096 |
| N75 | 8130 | 4638 | 9013 | 3135 | 3223 | 2180 | 9418 | 6270 | 12359 | 9647 | 12359 | 1627 | 4153 | 3951 | 1890 | 13340 | 16361 | 11191 | 8085 | 1158 | 4306 |
| L50 | 4 | 5 | 3 | 5 | 9 | 9 | 5 | 2 | 3 | 4 | 4 | 24 | 3 | 7 | 10 | 5 | 2 | 5 | 7 | 58 | 3 |
| L75 | 10 | 13 | 7 | 17 | 18 | 26 | 11 | 5 | 7 | 9 | 7 | 57 | 10 | 17 | 23 | 9 | 6 | 10 | 14 | 122 | 11 |
| # N's per 100 kbp | 0 | 0 | 0 | 0 | 0 | 0 | 0 | 0 | 0 | 0 | 0 | 0 | 0 | 0 | 0 | 0 | 0 | 0 | 0 | 0 | 0 |

**Supplementary Table 3: The table shows the genes of each sample with coverage, reads count, gene copy number, gene family, drug class, and resistance mechanism.**

| Sample | Gene Accession | Gene ID | Coverage | Reads Count | Gene Copy Number | AMR Gene Family | Drug Class | Resistance Mechanism |
|---|---|---|---|---|---|---|---|---|
| E1701 | AY044436 | CTX-M-15 | 100 | 30 | 0.034246575 | CTX-M beta-lactamase | cephalosporin | antibiotic inactivation |
| | AM886293.1 | rmtB | 100 | 23 | 0.03042328 | 16S rRNA methyltransferase (G1405) | aminoglycoside antibiotic | antibiotic target alteration |
| | KM998962.1 | TEM-220 | 91.05691057 | 46 | 0.053426249 | TEM beta-lactamase | monobactam;cephalosporin;penam;penem | antibiotic inactivation |
| | GU585907.1 | dfrA12 | 100 | 24 | 0.048192771 | trimethoprim resistant dihydrofolate reductase dfr | diaminopyrimidine antibiotic | antibiotic target replacement |
| | JF969163 | sul1 | 90.47619048 | 24 | 0.028571429 | sulfonamide resistant sul | sulfonamide antibiotic | antibiotic target replacement |
| | EU022314.1 | AAC(3)-IId | 100 | 30 | 0.034843206 | AAC(3) | aminoglycoside antibiotic | antibiotic inactivation |
| | D16251.1 | mphA | 99.66887417 | 29 | 0.03200883 | macrolide phosphotransferase (MPH) | macrolide antibiotic | antibiotic inactivation |
| | AF242872.1 | ErmB | 98.79518072 | 18 | 0.024096386 | Erm 23S ribosomal RNA methyltransferase | macrolide antibiotic;lincosamide antibiotic;streptogramin antibiotic | antibiotic target alteration |
| E1702 | AY055428.1 | sul2 | 88.60294118 | 9 | 0.011029412 | sulfonamide resistant sul | sulfonamide antibiotic | antibiotic target replacement |
| | JF969163 | sul1 | 100 | 43 | 0.051190476 | sulfonamide resistant sul | sulfonamide antibiotic | antibiotic target replacement |
| | AB089595 | tet(B) | 100 | 36 | 0.029850746 | major facilitator superfamily (MFS) antibiotic efflux pump | tetracycline antibiotic | antibiotic efflux |
| | DQ303918 | AAC(6')-Ib-cr | 100 | 8 | 0.013333333 | AAC(6') | fluoroquinolone antibiotic;aminoglycoside antibiotic | antibiotic inactivation |
| | AF137361 | aadA5 | 100 | 13 | 0.016476553 | ANT(3'') | aminoglycoside antibiotic | antibiotic inactivation |
| | GU585907.1 | dfrA12 | 100 | 23 | 0.046184739 | trimethoprim resistant dihydrofolate reductase dfr | diaminopyrimidine antibiotic | antibiotic target replacement |
| | D16251.1 | mphA | 92.93598234 | 14 | 0.015452539 | macrolide phosphotransferase (MPH) | macrolide antibiotic | antibiotic inactivation |
| | DQ838665 | dfrA17 | 100 | 11 | 0.023206751 | trimethoprim resistant dihydrofolate reductase dfr | diaminopyrimidine antibiotic | antibiotic target replacement |
| | AF024602 | APH(6)-Id | 100 | 12 | 0.014336918 | APH(6) | aminoglycoside antibiotic | antibiotic inactivation |
| | KR780479.1 | OXA-140 | 92.49291785 | 14 | 0.019830028 | OXA beta-lactamase | cephalosporin;penam | antibiotic inactivation |
| | AY044436 | CTX-M-15 | 86.64383562 | 7 | 0.007990868 | CTX-M beta-lactamase | cephalosporin | antibiotic inactivation |
| E1703 | GU585907.1 | dfrA12 | 100 | 28 | 0.0562249 | trimethoprim resistant dihydrofolate reductase dfr | diaminopyrimidine antibiotic | antibiotic target replacement |
| | JF969163 | sul1 | 96.54761905 | 16 | 0.019047619 | sulfonamide resistant sul | sulfonamide antibiotic | antibiotic target replacement |
| | AF024602 | APH(6)-Id | 97.61051374 | 12 | 0.014336918 | APH(6) | aminoglycoside antibiotic | antibiotic inactivation |
| | AY055428.1 | sul2 | 86.02941176 | 5 | 0.006127451 | sulfonamide resistant sul | sulfonamide antibiotic | antibiotic target replacement |
| | KM998962.1 | TEM-220 | 92.10220674 | 19 | 0.022067364 | TEM beta-lactamase | monobactam;cephalosporin;penam;penem | antibiotic inactivation |
| | AB089595 | tet(B) | 100 | 20 | 0.016583748 | major facilitator superfamily (MFS) antibiotic efflux pump | tetracycline antibiotic | antibiotic efflux |
| | KR780479.1 | OXA-140 | 93.05949008 | 14 | 0.019830028 | OXA beta-lactamase | cephalosporin;penam | antibiotic inactivation |
| | AY044436 | CTX-M-15 | 92.23744292 | 10 | 0.011415525 | CTX-M beta-lactamase | cephalosporin | antibiotic inactivation |
| E1704 | JF969163 | sul1 | 100 | 14 | 0.016666667 | sulfonamide resistant sul | sulfonamide antibiotic | antibiotic target replacement |
| | AM886293.1 | rmtB | 94.84126984 | 18 | 0.023809524 | 16S rRNA methyltransferase (G1405) | aminoglycoside antibiotic | antibiotic target alteration |
| | KM998962.1 | TEM-220 | 97.09639954 | 26 | 0.030197445 | TEM beta-lactamase | monobactam;cephalosporin;penam;penem | antibiotic inactivation |
| | EU022314.1 | AAC(3)-IId | 88.26945412 | 10 | 0.011614402 | AAC(3) | aminoglycoside antibiotic | antibiotic inactivation |
| | GU585907.1 | dfrA12 | 100 | 7 | 0.014056225 | trimethoprim resistant dihydrofolate reductase dfr | diaminopyrimidine antibiotic | antibiotic target replacement |
| E1705 | AY055428.1 | sul2 | 100 | 846 | 1.036764706 | sulfonamide resistant sul | sulfonamide antibiotic | antibiotic target replacement |
| | AF024602 | APH(6)-Id | 100 | 1284 | 1.534050179 | APH(6) | aminoglycoside antibiotic | antibiotic inactivation |
| | AF313472 | APH(3'')-Ib | 100 | 918 | 1.141791045 | APH(3'') | aminoglycoside antibiotic | antibiotic inactivation |
| | JF969163 | sul1 | 93.45238095 | 31 | 0.036904762 | sulfonamide resistant sul | sulfonamide antibiotic | antibiotic target replacement |
| | DQ838665 | dfrA17 | 100 | 11 | 0.023206751 | trimethoprim resistant dihydrofolate reductase dfr | diaminopyrimidine antibiotic | antibiotic target replacement |
| | AF534183.1 | tet(A) | 90.11764706 | 208 | 0.163137255 | major facilitator superfamily (MFS) antibiotic efflux pump | glycylcycline;tetracycline antibiotic | antibiotic efflux |
| | AM886293.1 | rmtB | 100 | 34 | 0.044973545 | 16S rRNA methyltransferase (G1405) | aminoglycoside antibiotic | antibiotic target alteration |
| | AF242872.1 | ErmB | 100 | 28 | 0.037483266 | Erm 23S ribosomal RNA methyltransferase | macrolide antibiotic;lincosamide antibiotic;streptogramin antibiotic | antibiotic target alteration |
| | D16251.1 | mphA | 100 | 31 | 0.034216336 | macrolide phosphotransferase (MPH) | macrolide antibiotic | antibiotic inactivation |
| | KM998962.1 | TEM-220 | 93.728223 | 20 | 0.023228804 | TEM beta-lactamase | monobactam;cephalosporin;penam;penem | antibiotic inactivation |
| | GU585907.1 | dfrA12 | 100 | 22 | 0.044176707 | trimethoprim resistant dihydrofolate reductase dfr | diaminopyrimidine antibiotic | antibiotic target replacement |
| | AY044436 | CTX-M-15 | 92.92237443 | 17 | 0.019406393 | CTX-M beta-lactamase | cephalosporin | antibiotic inactivation |
| | DQ303918 | AAC(6')-Ib-cr | 100 | 11 | 0.018333333 | AAC(6') | fluoroquinolone antibiotic;aminoglycoside antibiotic | antibiotic inactivation |
| E1706 | AF024602 | APH(6)-Id | 100 | 526 | 0.628434886 | APH(6) | aminoglycoside antibiotic | antibiotic inactivation |
| | AY055428.1 | sul2 | 100 | 394 | 0.482843137 | sulfonamide resistant sul | sulfonamide antibiotic | antibiotic target replacement |
| | AF313472 | APH(3'')-Ib | 100 | 406 | 0.504975124 | APH(3'') | aminoglycoside antibiotic | antibiotic inactivation |
| | EU022314.1 | AAC(3)-IId | 100 | 22 | 0.025551684 | AAC(3) | aminoglycoside antibiotic | antibiotic inactivation |
| | AM886293.1 | rmtB | 86.77248677 | 7 | 0.009259259 | 16S rRNA methyltransferase (G1405) | aminoglycoside antibiotic | antibiotic target alteration |
| | AY044436 | CTX-M-15 | 100 | 25 | 0.028538813 | CTX-M beta-lactamase | cephalosporin | antibiotic inactivation |
| | KM998962.1 | TEM-220 | 96.05110337 | 37 | 0.042973287 | TEM beta-lactamase | monobactam;cephalosporin;penam;penem | antibiotic inactivation |
| | GU585907.1 | dfrA12 | 93.97590361 | 7 | 0.014056225 | trimethoprim resistant dihydrofolate reductase dfr | diaminopyrimidine antibiotic | antibiotic target replacement |
| | AF242872.1 | ErmB | 85.27443106 | 7 | 0.009370817 | Erm 23S ribosomal RNA methyltransferase | macrolide antibiotic;lincosamide antibiotic;streptogramin antibiotic | antibiotic target alteration |
| E1707 | KM998962.1 | TEM-220 | 90.82462253 | 77 | 0.089430894 | TEM beta-lactamase | monobactam;cephalosporin;penam;penem | antibiotic inactivation |
| | JF969163 | sul1 | 100 | 47 | 0.055952381 | sulfonamide resistant sul | sulfonamide antibiotic | antibiotic target replacement |
| | D16251.1 | mphA | 99.66887417 | 35 | 0.038631347 | macrolide phosphotransferase (MPH) | macrolide antibiotic | antibiotic inactivation |
| | AM886293.1 | rmtB | 98.14814815 | 28 | 0.037037037 | 16S rRNA methyltransferase (G1405) | aminoglycoside antibiotic | antibiotic target alteration |
| | AY044436 | CTX-M-15 | 100 | 33 | 0.037671233 | CTX-M beta-lactamase | cephalosporin | antibiotic inactivation |
| | GU585907.1 | dfrA12 | 100 | 47 | 0.09437751 | trimethoprim resistant dihydrofolate reductase dfr | diaminopyrimidine antibiotic | antibiotic target replacement |
| | AF242872.1 | ErmB | 100 | 21 | 0.02811245 | Erm 23S ribosomal RNA methyltransferase | macrolide antibiotic;lincosamide antibiotic;streptogramin antibiotic | antibiotic target alteration |
| | EU022314.1 | AAC(3)-IId | 100 | 31 | 0.036004646 | AAC(3) | aminoglycoside antibiotic | antibiotic inactivation |

| | | | | | | | | |
|---|---|---|---|---|---|---|---|---|
| E1708 | AY055428.1 | sul2 | 100 | 1191 | 1.459558824 | sulfonamide resistant sul | sulfonamide antibiotic | antibiotic target replacement |
| | AF313472 | APH(3")-Ib | 100 | 1184 | 1.472636816 | APH(3") | aminoglycoside antibiotic | antibiotic inactivation |
| | KM998962.1 | TEM-220 | 98.02555168 | 150 | 0.174216028 | TEM beta-lactamase | monobactam;cephalosporin;penam;penem | antibiotic inactivation |
| | AF024602 | APH(6)-Id | 100 | 1574 | 1.880525687 | APH(6) | aminoglycoside antibiotic | antibiotic inactivation |
| | JF969163 | sul1 | 100 | 54 | 0.064285714 | sulfonamide resistant sul | sulfonamide antibiotic | antibiotic target replacement |
| | AY044436 | CTX-M-15 | 100 | 141 | 0.160958904 | CTX-M beta-lactamase | cephalosporin | antibiotic inactivation |
| | D16251.1 | mphA | 99.66887417 | 31 | 0.034216336 | macrolide phosphotransferase (MPH) | macrolide antibiotic | antibiotic inactivation |
| | DQ838665 | dfrA17 | 100 | 25 | 0.052742616 | trimethoprim resistant dihydrofolate reductase dfr | diaminopyrimidine antibiotic | antibiotic target replacement |
| | AF137361 | aadA5 | 100 | 25 | 0.031685678 | ANT(3") | aminoglycoside antibiotic | antibiotic inactivation |
| | AM886293.1 | rmtB | 100 | 33 | 0.043650794 | 16S rRNA methyltransferase (G1405) | aminoglycoside antibiotic | antibiotic target alteration |
| | GU585907.1 | dfrA12 | 100 | 20 | 0.040160643 | trimethoprim resistant dihydrofolate reductase dfr | diaminopyrimidine antibiotic | antibiotic target replacement |
| | KP265939.1 | NDM-11 | 86.96186962 | 14 | 0.017220172 | NDM beta-lactamase | carbapenem;cephalosporin;cephamycin;penam | antibiotic inactivation |
| | AF242872.1 | ErmB | 100 | 21 | 0.02811245 | Erm 23S ribosomal RNA methyltransferase | macrolide antibiotic;lincosamide antibiotic;streptogramin antibiotic | antibiotic target alteration |
| E1709 | EU022314.1 | AAC(3)-IId | 100 | 25 | 0.029036005 | AAC(3) | aminoglycoside antibiotic | antibiotic inactivation |
| | JF969163 | sul1 | 100 | 106 | 0.126190476 | sulfonamide resistant sul | sulfonamide antibiotic | antibiotic target replacement |
| | AF242872.1 | ErmB | 94.24364123 | 12 | 0.016064257 | Erm 23S ribosomal RNA methyltransferase | macrolide antibiotic;lincosamide antibiotic;streptogramin antibiotic | antibiotic target alteration |
| | KP265939.1 | NDM-11 | 95.0799508 | 66 | 0.081180812 | NDM beta-lactamase | carbapenem;cephalosporin;cephamycin;penam | antibiotic inactivation |
| | KM998962.1 | TEM-220 | 97.90940767 | 67 | 0.077816492 | TEM beta-lactamase | monobactam;cephalosporin;penam;penem | antibiotic inactivation |
| | GU585907.1 | dfrA12 | 100 | 92 | 0.184738956 | trimethoprim resistant dihydrofolate reductase dfr | diaminopyrimidine antibiotic | antibiotic target replacement |
| | D16251.1 | mphA | 99.66887417 | 37 | 0.040838852 | macrolide phosphotransferase (MPH) | macrolide antibiotic | antibiotic inactivation |
| | AM886293.1 | rmtB | 100 | 26 | 0.034391534 | 16S rRNA methyltransferase (G1405) | aminoglycoside antibiotic | antibiotic target alteration |
| | AY044436 | CTX-M-15 | 100 | 49 | 0.055936073 | CTX-M beta-lactamase | cephalosporin | antibiotic inactivation |
| | JQ394987 | mdfA | 89.13219789 | 8 | 0.00648824 | major facilitator superfamily (MFS) antibiotic efflux pump | tetracycline antibiotic;benzalkonium chloride;rhodamine | antibiotic efflux |
| E1710 | JF969163 | sul1 | 100 | 93 | 0.110714286 | sulfonamide resistant sul | sulfonamide antibiotic | antibiotic target replacement |
| | GU585907.1 | dfrA12 | 100 | 71 | 0.142570281 | trimethoprim resistant dihydrofolate reductase dfr | diaminopyrimidine antibiotic | antibiotic target replacement |
| | AY044436 | CTX-M-15 | 100 | 66 | 0.075342466 | CTX-M beta-lactamase | cephalosporin | antibiotic inactivation |
| | EU022314.1 | AAC(3)-IId | 100 | 41 | 0.047619048 | AAC(3) | aminoglycoside antibiotic | antibiotic inactivation |
| | AM886293.1 | rmtB | 100 | 37 | 0.048941799 | 16S rRNA methyltransferase (G1405) | aminoglycoside antibiotic | antibiotic target alteration |
| | KM998962.1 | TEM-220 | 96.28339141 | 80 | 0.092915215 | TEM beta-lactamase | monobactam;cephalosporin;penam;penem | antibiotic inactivation |
| | KP265939.1 | NDM-11 | 97.04797048 | 39 | 0.04797048 | NDM beta-lactamase | carbapenem;cephalosporin;cephamycin;penam | antibiotic inactivation |
| | D16251.1 | mphA | 99.66887417 | 45 | 0.049668874 | macrolide phosphotransferase (MPH) | macrolide antibiotic | antibiotic inactivation |
| | AF242872.1 | ErmB | 91.83400268 | 13 | 0.017402945 | Erm 23S ribosomal RNA methyltransferase | macrolide antibiotic;lincosamide antibiotic;streptogramin antibiotic | antibiotic target alteration |
| E1711 | KM998962.1 | TEM-220 | 96.05110337 | 112 | 0.130081301 | TEM beta-lactamase | monobactam;cephalosporin;penam;penem | antibiotic inactivation |
| | KP265939.1 | NDM-11 | 97.5399754 | 53 | 0.065190652 | NDM beta-lactamase | carbapenem;cephalosporin;cephamycin;penam | antibiotic inactivation |
| | GU585907.1 | dfrA12 | 100 | 76 | 0.152610442 | trimethoprim resistant dihydrofolate reductase dfr | diaminopyrimidine antibiotic | antibiotic target replacement |
| | JF969163 | sul1 | 100 | 82 | 0.097619048 | sulfonamide resistant sul | sulfonamide antibiotic | antibiotic target replacement |
| | EU022314.1 | AAC(3)-IId | 100 | 56 | 0.06504065 | AAC(3) | aminoglycoside antibiotic | antibiotic inactivation |
| | D16251.1 | mphA | 99.66887417 | 47 | 0.05187638 | macrolide phosphotransferase (MPH) | macrolide antibiotic | antibiotic inactivation |
| | AM040708.1 | aadA8b | 92.67676768 | 71 | 0.089646465 | ANT(3") | aminoglycoside antibiotic | antibiotic inactivation |
| | AM886293.1 | rmtB | 100 | 41 | 0.054232804 | 16S rRNA methyltransferase (G1405) | aminoglycoside antibiotic | antibiotic target alteration |
| | AF242872.1 | ErmB | 97.05488621 | 20 | 0.026773762 | Erm 23S ribosomal RNA methyltransferase | macrolide antibiotic;lincosamide antibiotic;streptogramin antibiotic | antibiotic target alteration |
| | AY044436 | CTX-M-15 | 100 | 56 | 0.063926941 | CTX-M beta-lactamase | cephalosporin | antibiotic inactivation |
| E1712 | AF024602 | APH(6)-Id | 100 | 763 | 0.911589008 | APH(6) | aminoglycoside antibiotic | antibiotic inactivation |
| | AY055428.1 | sul2 | 100 | 537 | 0.658088235 | sulfonamide resistant sul | sulfonamide antibiotic | antibiotic target replacement |
| | JF969163 | sul1 | 100 | 27 | 0.032142857 | sulfonamide resistant sul | sulfonamide antibiotic | antibiotic target replacement |
| | AF313472 | APH(3")-Ib | 100 | 555 | 0.690298507 | APH(3") | aminoglycoside antibiotic | antibiotic inactivation |
| | KM998962.1 | TEM-220 | 89.89547038 | 20 | 0.023228804 | TEM beta-lactamase | monobactam;cephalosporin;penam;penem | antibiotic inactivation |
| | GU585907.1 | dfrA12 | 100 | 16 | 0.032128514 | trimethoprim resistant dihydrofolate reductase dfr | diaminopyrimidine antibiotic | antibiotic target replacement |
| | AB089595 | tet(B) | 100 | 19 | 0.015754561 | major facilitator superfamily (MFS) antibiotic efflux pump | tetracycline antibiotic | antibiotic efflux |
| | U00096.3 | yojI | 90.1459854 | 16 | 0.00973236 | ATP-binding cassette (ABC) antibiotic efflux pump | peptide antibiotic | antibiotic efflux |
| | AM886293.1 | rmtB | 86.64021164 | 7 | 0.009259259 | 16S rRNA methyltransferase (G1405) | aminoglycoside antibiotic | antibiotic target alteration |
| | EU022314.1 | AAC(3)-IId | 96.63182346 | 8 | 0.009291521 | AAC(3) | aminoglycoside antibiotic | antibiotic inactivation |
| | AY044436 | CTX-M-15 | 88.47031963 | 10 | 0.011415525 | CTX-M beta-lactamase | cephalosporin | antibiotic inactivation |
| | JQ394987 | mdfA | 96.99918897 | 19 | 0.01540957 | major facilitator superfamily (MFS) antibiotic efflux pump | tetracycline antibiotic;benzalkonium chloride;rhodamine | antibiotic efflux |
| E1713 | AM886293.1 | rmtB | 100 | 44 | 0.058201058 | 16S rRNA methyltransferase (G1405) | aminoglycoside antibiotic | antibiotic target alteration |
| | AF242872.1 | ErmB | 100 | 45 | 0.060240964 | Erm 23S ribosomal RNA methyltransferase | macrolide antibiotic;lincosamide antibiotic;streptogramin antibiotic | antibiotic target alteration |
| | GU585907.1 | dfrA12 | 100 | 24 | 0.048192771 | trimethoprim resistant dihydrofolate reductase dfr | diaminopyrimidine antibiotic | antibiotic target replacement |
| | KM998962.1 | TEM-220 | 95.81881533 | 60 | 0.069686411 | TEM beta-lactamase | monobactam;cephalosporin;penam;penem | antibiotic inactivation |
| | AY055428.1 | sul2 | 96.44607843 | 14 | 0.017156863 | sulfonamide resistant sul | sulfonamide antibiotic | antibiotic target replacement |
| | AB089595 | tet(B) | 100 | 31 | 0.025704809 | major facilitator superfamily (MFS) antibiotic efflux pump | tetracycline antibiotic | antibiotic efflux |
| | JF969163 | sul1 | 100 | 31 | 0.036904762 | sulfonamide resistant sul | sulfonamide antibiotic | antibiotic target replacement |
| | KP265939.1 | NDM-11 | 85.2398524 | 21 | 0.025830258 | NDM beta-lactamase | carbapenem;cephalosporin;cephamycin;penam | antibiotic inactivation |
| | AF313472 | APH(3")-Ib | 100 | 10 | 0.012437811 | APH(3") | aminoglycoside antibiotic | antibiotic inactivation |
| | D16251.1 | mphA | 99.66887417 | 51 | 0.056291391 | macrolide phosphotransferase (MPH) | macrolide antibiotic | antibiotic inactivation |
| | AF137361 | aadA5 | 100 | 12 | 0.015209125 | ANT(3") | aminoglycoside antibiotic | antibiotic inactivation |
| | DQ838665 | dfrA17 | 87.55274262 | 6 | 0.012658228 | trimethoprim resistant dihydrofolate reductase dfr | diaminopyrimidine antibiotic | antibiotic target replacement |
| | AF024602 | APH(6)-Id | 100 | 20 | 0.023894863 | APH(6) | aminoglycoside antibiotic | antibiotic inactivation |
| | Z11877.1 | EB | 100 | 4 | 0.012012012 | small multidrug resistance (SMR) antibiotic efflux pump | macrolide antibiotic | antibiotic efflux |
| E1714 | JF969163 | sul1 | 100 | 44 | 0.052380952 | sulfonamide resistant sul | sulfonamide antibiotic | antibiotic target replacement |
| | AF137361 | aadA5 | 100 | 17 | 0.021546261 | ANT(3") | aminoglycoside antibiotic | antibiotic inactivation |
| | BX664015.1 | APH(3')-Ia | 99.75490196 | 17 | 0.020833333 | APH(3') | aminoglycoside antibiotic | antibiotic inactivation |
| | KP265939.1 | NDM-11 | 92.25092251 | 27 | 0.033210332 | NDM beta-lactamase | carbapenem;cephalosporin;cephamycin;penam | antibiotic inactivation |
| | AF078527 | arr-2 | 100 | 23 | 0.050772627 | rifampin ADP-ribosyltransferase (Arr) | rifamycin antibiotic | antibiotic inactivation |
| | GU585907.1 | dfrA12 | 100 | 34 | 0.068273092 | trimethoprim resistant dihydrofolate reductase dfr | diaminopyrimidine antibiotic | antibiotic target replacement |
| | FJ196385 | sul3 | 100 | 22 | 0.027777778 | sulfonamide resistant sul | sulfonamide antibiotic | antibiotic target replacement |
| | AF024602 | APH(6)-Id | 100 | 16 | 0.01911589 | APH(6) | aminoglycoside antibiotic | antibiotic inactivation |
| | DQ838665 | dfrA17 | 100 | 10 | 0.021097046 | trimethoprim resistant dihydrofolate reductase dfr | diaminopyrimidine antibiotic | antibiotic target replacement |
| | KM998962.1 | TEM-220 | 96.39953542 | 68 | 0.078977933 | TEM beta-lactamase | monobactam;cephalosporin;penam;penem | antibiotic inactivation |
| | AM886293.1 | rmtB | 100 | 38 | 0.05026455 | 16S rRNA methyltransferase (G1405) | aminoglycoside antibiotic | antibiotic target alteration |
| | D16251.1 | mphA | 100 | 52 | 0.057395143 | macrolide phosphotransferase (MPH) | macrolide antibiotic | antibiotic inactivation |
| | AF231986 | floR | 98.51851852 | 25 | 0.020576132 | major facilitator superfamily (MFS) antibiotic efflux pump | phenicol antibiotic | antibiotic efflux |
| | MK303617.1 | QnrS15 | 98.17351598 | 17 | 0.02587519 | quinolone resistance protein (qnr) | fluoroquinolone antibiotic | antibiotic target protection |
| | JCMW02000002.1 | OXA-663 | 92.88389513 | 15 | 0.018726592 | OXA beta-lactamase | cephalosporin;penam | antibiotic inactivation |
| | AF313472 | APH(3")-Ib | 90.29850746 | 15 | 0.018656716 | APH(3") | aminoglycoside antibiotic | antibiotic inactivation |

| | | | | | | | | |
|---|---|---|---|---|---|---|---|---|
| E1715 | GU585907.1 | dfrA12 | 100 | 153 | 0.307228916 | trimethoprim resistant dihydrofolate reductase dfr | diaminopyrimidine antibiotic | antibiotic target replacement |
| | AM886293.1 | rmtB | 100 | 217 | 0.287037037 | 16S rRNA methyltransferase (G1405) | aminoglycoside antibiotic | antibiotic target alteration |
| | KM998962.1 | TEM-220 | 98.49012776 | 277 | 0.321718931 | TEM beta-lactamase | monobactam;cephalosporin;penam;penem | antibiotic inactivation |
| | D16251.1 | mphA | 99.66887417 | 83 | 0.091611479 | macrolide phosphotransferase (MPH) | macrolide antibiotic | antibiotic inactivation |
| | KP265939.1 | NDM-11 | 95.32595326 | 115 | 0.141451415 | NDM beta-lactamase | carbapenem;cephalosporin;cephamycin;penam | antibiotic inactivation |
| | AF242872.1 | ErmB | 100 | 54 | 0.072289157 | Erm 23S ribosomal RNA methyltransferase | macrolide antibiotic;lincosamide antibiotic;streptogramin antibiotic | antibiotic target alteration |
| | JF969163 | sul1 | 100 | 215 | 0.255952381 | sulfonamide resistant sul | sulfonamide antibiotic | antibiotic target replacement |
| E1716 | AF024602 | APH(6)-Id | 100 | 957 | 1.143369176 | APH(6) | aminoglycoside antibiotic | antibiotic inactivation |
| | AF313472 | APH(3'')-Ib | 100 | 654 | 0.813432836 | APH(3'') | aminoglycoside antibiotic | antibiotic inactivation |
| | AY055428.1 | sul2 | 100 | 713 | 0.87377451 | sulfonamide resistant sul | sulfonamide antibiotic | antibiotic target replacement |
| | D16251.1 | mphA | 97.01986755 | 13 | 0.014348786 | macrolide phosphotransferase (MPH) | macrolide antibiotic | antibiotic inactivation |
| | AM886293.1 | rmtB | 98.67724868 | 19 | 0.025132275 | 16S rRNA methyltransferase (G1405) | aminoglycoside antibiotic | antibiotic target alteration |
| | GU585907.1 | dfrA12 | 100 | 11 | 0.022088353 | trimethoprim resistant dihydrofolate reductase dfr | diaminopyrimidine antibiotic | antibiotic target replacement |
| | AF231986 | floR | 100 | 19 | 0.01563786 | major facilitator superfamily (MFS) antibiotic efflux pump | phenicol antibiotic | antibiotic efflux |
| | KM998962.1 | TEM-220 | 92.68292683 | 18 | 0.020905923 | TEM beta-lactamase | monobactam;cephalosporin;penam;penem | antibiotic inactivation |
| | AF242872.1 | ErmB | 100 | 21 | 0.02811245 | Erm 23S ribosomal RNA methyltransferase | macrolide antibiotic;lincosamide antibiotic;streptogramin antibiotic | antibiotic target alteration |
| E1717 | AF024602 | APH(6)-Id | 100 | 31 | 0.037037037 | APH(6) | aminoglycoside antibiotic | antibiotic inactivation |
| | JF969163 | sul1 | 100 | 54 | 0.064285714 | sulfonamide resistant sul | sulfonamide antibiotic | antibiotic target replacement |
| | D16251.1 | mphA | 99.66887417 | 57 | 0.062913907 | macrolide phosphotransferase (MPH) | macrolide antibiotic | antibiotic inactivation |
| | AF534183.1 | tet(A) | 100 | 158 | 0.123921569 | major facilitator superfamily (MFS) antibiotic efflux pump | glycylcycline;tetracycline antibiotic | antibiotic efflux |
| | MK303617.1 | QnrS15 | 89.49771689 | 15 | 0.02283105 | quinolone resistance protein (qnr) | fluoroquinolone antibiotic | antibiotic target protection |
| | KP265939.1 | NDM-11 | 95.57195572 | 53 | 0.065190652 | NDM beta-lactamase | carbapenem;cephalosporin;cephamycin;penam | antibiotic inactivation |
| | AY044436 | CTX-M-15 | 100 | 30 | 0.034246575 | CTX-M beta-lactamase | cephalosporin | antibiotic inactivation |
| | AF313472 | APH(3'')-Ib | 100 | 15 | 0.018656716 | APH(3'') | aminoglycoside antibiotic | antibiotic inactivation |
| | KM998962.1 | TEM-220 | 95.12195122 | 67 | 0.077816492 | TEM beta-lactamase | monobactam;cephalosporin;penam;penem | antibiotic inactivation |
| | AF242872.1 | ErmB | 100 | 28 | 0.037483266 | Erm 23S ribosomal RNA methyltransferase | macrolide antibiotic;lincosamide antibiotic;streptogramin antibiotic | antibiotic target alteration |
| | AY055428.1 | sul2 | 92.76960784 | 17 | 0.020833333 | sulfonamide resistant sul | sulfonamide antibiotic | antibiotic target replacement |
| | AM886293.1 | rmtB | 100 | 52 | 0.068783069 | 16S rRNA methyltransferase (G1405) | aminoglycoside antibiotic | antibiotic target alteration |
| | GU585907.1 | dfrA12 | 100 | 33 | 0.06626506 | trimethoprim resistant dihydrofolate reductase dfr | diaminopyrimidine antibiotic | antibiotic target replacement |
| E1718 | AM886293.1 | rmtB | 100 | 33 | 0.043650794 | 16S rRNA methyltransferase (G1405) | aminoglycoside antibiotic | antibiotic target alteration |
| | KM998962.1 | TEM-220 | 98.25783972 | 47 | 0.054587689 | TEM beta-lactamase | monobactam;cephalosporin;penam;penem | antibiotic inactivation |
| | D16251.1 | mphA | 99.66887417 | 51 | 0.056291391 | macrolide phosphotransferase (MPH) | macrolide antibiotic | antibiotic inactivation |
| | DQ278190 | dfrA5 | 100 | 27 | 0.056962025 | trimethoprim resistant dihydrofolate reductase dfr | diaminopyrimidine antibiotic | antibiotic target replacement |
| | AF242872.1 | ErmB | 100 | 40 | 0.053547523 | Erm 23S ribosomal RNA methyltransferase | macrolide antibiotic;lincosamide antibiotic;streptogramin antibiotic | antibiotic target alteration |
| | JF969163 | sul1 | 100 | 59 | 0.070238095 | sulfonamide resistant sul | sulfonamide antibiotic | antibiotic target replacement |
| | GU585907.1 | dfrA12 | 100 | 30 | 0.060240964 | trimethoprim resistant dihydrofolate reductase dfr | diaminopyrimidine antibiotic | antibiotic target replacement |
| | AY044436 | CTX-M-15 | 100 | 24 | 0.02739726 | CTX-M beta-lactamase | cephalosporin | antibiotic inactivation |
| | AF024602 | APH(6)-Id | 100 | 20 | 0.023894863 | APH(6) | aminoglycoside antibiotic | antibiotic inactivation |
| | AY055428.1 | sul2 | 99.87745098 | 13 | 0.015931373 | sulfonamide resistant sul | sulfonamide antibiotic | antibiotic target replacement |
| | AF534183.1 | tet(A) | 99.76470588 | 25 | 0.019607843 | major facilitator superfamily (MFS) antibiotic efflux pump | glycylcycline;tetracycline antibiotic | antibiotic efflux |
| | KP265939.1 | NDM-11 | 87.82287823 | 32 | 0.039360394 | NDM beta-lactamase | carbapenem;cephalosporin;cephamycin;penam | antibiotic inactivation |
| | MK303617.1 | QnrS15 | 89.04109589 | 37 | 0.056316591 | quinolone resistance protein (qnr) | fluoroquinolone antibiotic | antibiotic target protection |
| E1719 | AF024602 | APH(6)-Id | 100 | 24 | 0.028673835 | APH(6) | aminoglycoside antibiotic | antibiotic inactivation |
| | D16251.1 | mphA | 99.66887417 | 48 | 0.052980132 | macrolide phosphotransferase (MPH) | macrolide antibiotic | antibiotic inactivation |
| | KM998962.1 | TEM-220 | 94.88966318 | 56 | 0.06504065 | TEM beta-lactamase | monobactam;cephalosporin;penam;penem | antibiotic inactivation |
| | JF969163 | sul1 | 100 | 42 | 0.05 | sulfonamide resistant sul | sulfonamide antibiotic | antibiotic target replacement |
| | KP265939.1 | NDM-11 | 89.79089791 | 43 | 0.052890529 | NDM beta-lactamase | carbapenem;cephalosporin;cephamycin;penam | antibiotic inactivation |
| | AF534183.1 | tet(A) | 100 | 71 | 0.055686275 | major facilitator superfamily (MFS) antibiotic efflux pump | glycylcycline;tetracycline antibiotic | antibiotic efflux |
| | AM886293.1 | rmtB | 100 | 23 | 0.03042328 | 16S rRNA methyltransferase (G1405) | aminoglycoside antibiotic | antibiotic target alteration |
| | DQ278190 | dfrA5 | 100 | 21 | 0.044303797 | trimethoprim resistant dihydrofolate reductase dfr | diaminopyrimidine antibiotic | antibiotic target replacement |
| | MK303617.1 | QnrS15 | 99.23896499 | 31 | 0.04718417 | quinolone resistance protein (qnr) | fluoroquinolone antibiotic | antibiotic target protection |
| | AF242872.1 | ErmB | 100 | 39 | 0.052208835 | Erm 23S ribosomal RNA methyltransferase | macrolide antibiotic;lincosamide antibiotic;streptogramin antibiotic | antibiotic target alteration |
| | AY055428.1 | sul2 | 100 | 15 | 0.018382353 | sulfonamide resistant sul | sulfonamide antibiotic | antibiotic target replacement |
| | AY044436 | CTX-M-15 | 100 | 18 | 0.020547945 | CTX-M beta-lactamase | cephalosporin | antibiotic inactivation |
| | GU585907.1 | dfrA12 | 100 | 25 | 0.050200803 | trimethoprim resistant dihydrofolate reductase dfr | diaminopyrimidine antibiotic | antibiotic target replacement |
| E1720 | AF024602 | APH(6)-Id | 100 | 531 | 0.634408602 | APH(6) | aminoglycoside antibiotic | antibiotic inactivation |
| | AY055428.1 | sul2 | 100 | 413 | 0.506127451 | sulfonamide resistant sul | sulfonamide antibiotic | antibiotic target replacement |
| | AF313472 | APH(3'')-Ib | 100 | 370 | 0.460199005 | APH(3'') | aminoglycoside antibiotic | antibiotic inactivation |
| | MK303617.1 | QnrS15 | 97.56468798 | 7 | 0.01065449 | quinolone resistance protein (qnr) | fluoroquinolone antibiotic | antibiotic target protection |
| | KR401105.1 | OXA-484 | 92.73182957 | 16 | 0.020050125 | OXA beta-lactamase | cephalosporin;penam | antibiotic inactivation |
| E1721 | AF231986 | floR | 100 | 51 | 0.041975309 | major facilitator superfamily (MFS) antibiotic efflux pump | phenicol antibiotic | antibiotic efflux |
| | EU780012 | dfrA14 | 100 | 37 | 0.076604555 | trimethoprim resistant dihydrofolate reductase dfr | diaminopyrimidine antibiotic | antibiotic target replacement |
| | AF534183.1 | tet(A) | 100 | 67 | 0.05254902 | major facilitator superfamily (MFS) antibiotic efflux pump | glycylcycline;tetracycline antibiotic | antibiotic efflux |
| | BX664015.1 | APH(3')-Ia | 100 | 48 | 0.058823529 | APH(3') | aminoglycoside antibiotic | antibiotic inactivation |
| | DQ303918 | AAC(6')-Ib-cr | 100 | 10 | 0.016666667 | AAC(6') | fluoroquinolone antibiotic;aminoglycoside antibiotic | antibiotic inactivation |
| | MK303617.1 | QnrS15 | 95.43378995 | 29 | 0.04414003 | quinolone resistance protein (qnr) | fluoroquinolone antibiotic | antibiotic target protection |
| | GU585907.1 | dfrA12 | 92.36947791 | 8 | 0.016064257 | trimethoprim resistant dihydrofolate reductase dfr | diaminopyrimidine antibiotic | antibiotic target replacement |
| | AE005174.2 | mphB | 93.29140461 | 2 | 0.004192872 | macrolide phosphotransferase (MPH) | macrolide antibiotic | antibiotic inactivation |

**Supplementary Table 4: The table shows the plasmids of each sample with coverage, contigs count, plasmid copy number, and source organism.**

| Sample | Plasmid ID | Plasmid Accession | Coverage | Contigs Count | Plasmid Copy Number | Source Organism |
|---|---|---|---|---|---|---|
| E1701 | NZ_CP014494 (pMVAST0167_2) | NZ_CP014494.1 | 90.76108973 | 14 | 0.000177233 | Escherichia coli |
| | NZ_CP010879 (pMNCRE44_3) | NZ_CP010879.1 | 100 | 2 | 0.000387072 | Escherichia coli |
| | NC_009347 (pSS046_spC) | NC_009347.1 | 100 | 2 | 0.000951928 | Shigella sonnei |
| | NZ_CP018439 (pKp_Goe_917-8) | NZ_CP018439.1 | 100 | 2 | 0.001034661 | Klebsiella pneumoniae |
| | NZ_CP011143 (pVR50I) | NZ_CP011143.1 | 98.7757732 | 1 | 0.00064433 | Escherichia coli |
| E1702 | NC_016824 (plasmid C) | NC_016824.1 | 100 | 2 | 0.000957396 | Shigella sonnei |
| | NC_018652 (pG-09EL50) | NC_018652.1 | 99.09619109 | 2 | 0.001291156 | Escherichia coli |
| E1703 | NC_016824 (plasmid C) | NC_016824.1 | 100 | 2 | 0.000957396 | Shigella sonnei |
| | NZ_CP015143 (pEC732_5) | NZ_CP015143.1 | 100 | 2 | 0.001291156 | Escherichia coli |
| E1704 | NZ_CP010879 (pMNCRE44_3) | NZ_CP010879.1 | 100 | 2 | 0.000387072 | Escherichia coli |
| | NC_016824 (plasmid C) | NC_016824.1 | 100 | 2 | 0.000957396 | Shigella sonnei |
| | NZ_CP011143 (pVR50I) | NZ_CP011143.1 | 98.7757732 | 1 | 0.00064433 | Escherichia coli |
| E1706 | NZ_CP006639 (PCN061p3) | NZ_CP006639.1 | 96.46415943 | 2 | 0.00032144 | Escherichia coli |
| E1707 | NZ_CP019692 (p75-02_4) | NZ_CP019692.1 | 100 | 3 | 0.000473111 | Shigella sonnei |
| | NZ_CP010879 (pMNCRE44_3) | NZ_CP010879.1 | 100 | 2 | 0.000387072 | Escherichia coli |
| | NC_016824 (plasmid C) | NC_016824.1 | 100 | 2 | 0.000957396 | Shigella sonnei |
| | NZ_CP018439 (pKp_Goe_917-8) | NZ_CP018439.1 | 100 | 2 | 0.001034661 | Klebsiella pneumoniae |
| | NZ_CP011143 (pVR50I) | NZ_CP011143.1 | 98.7757732 | 1 | 0.00064433 | Escherichia coli |
| E1709 | NZ_CP010879 (pMNCRE44_3) | NZ_CP010879.1 | 100 | 2 | 0.000387072 | Escherichia coli |
| | NC_009347 (pSS046_spC) | NC_009347.1 | 100 | 2 | 0.000951928 | Shigella sonnei |
| | NZ_CP018439 (pKp_Goe_917-8) | NZ_CP018439.1 | 100 | 2 | 0.001034661 | Klebsiella pneumoniae |
| | NZ_CP011143 (pVR50I) | NZ_CP011143.1 | 98.7757732 | 1 | 0.00064433 | Escherichia coli |
| E1710 | NZ_CP010879 (pMNCRE44_3) | NZ_CP010879.1 | 100 | 2 | 0.000387072 | Escherichia coli |
| | NC_009347 (pSS046_spC) | NC_009347.1 | 97.95335554 | 1 | 0.000475964 | Shigella sonnei |
| | NZ_CP018439 (pKp_Goe_917-8) | NZ_CP018439.1 | 100 | 2 | 0.001034661 | Klebsiella pneumoniae |
| | NZ_CP011143 (pVR50I) | NZ_CP011143.1 | 98.7757732 | 1 | 0.00064433 | Escherichia coli |
| E1711 | NZ_CP010879 (pMNCRE44_3) | NZ_CP010879.1 | 100 | 2 | 0.000387072 | Escherichia coli |
| | NC_009347 (pSS046_spC) | NC_009347.1 | 100 | 2 | 0.000951928 | Shigella sonnei |
| | NZ_CP018439 (pKp_Goe_917-8) | NZ_CP018439.1 | 100 | 2 | 0.001034661 | Klebsiella pneumoniae |
| | NZ_CP011143 (pVR50I) | NZ_CP011143.1 | 98.7757732 | 1 | 0.00064433 | Escherichia coli |
| E1712 | NZ_CP006639 (PCN061p3) | NZ_CP006639.1 | 100 | 2 | 0.00032144 | Escherichia coli |
| | NZ_CP013225 (PDM05) | NZ_CP013225.1 | 100 | 2 | 0.000593296 | Salmonella enterica |
| E1713 | NZ_CP013223 (PDM03) | NZ_CP013223.1 | 99.94067042 | 2 | 0.000593296 | Salmonella enterica |
| E1715 | NZ_CP010879 (pMNCRE44_3) | NZ_CP010879.1 | 100 | 2 | 0.000387072 | Escherichia coli |
| | NC_016824 (plasmid C) | NC_016824.1 | 100 | 2 | 0.000957396 | Shigella sonnei |
| | NZ_CP011143 (pVR50I) | NZ_CP011143.1 | 98.7757732 | 1 | 0.00064433 | Escherichia coli |
| E1716 | NC_017329 (pSFxv_3) | NC_017329.1 | 98.74193548 | 2 | 0.000322581 | Shigella flexneri |
| | NZ_CP008719 (pEC648_5) | NZ_CP008719.1 | 100 | 2 | 0.000951928 | Escherichia coli |
| E1717 | NZ_LN890525 (Plasmid 2) | NZ_LN890525.1 | 99.45050419 | 17 | 0.000200031 | Salmonella enterica |
| | NZ_CP008719 (pEC648_5) | NZ_CP008719.1 | 100 | 2 | 0.000951928 | Escherichia coli |
| E1718 | NC_016824 (plasmid C) | NC_016824.1 | 100 | 2 | 0.000957396 | Shigella sonnei |
| E1719 | NC_016824 (plasmid C) | NC_016824.1 | 100 | 2 | 0.000957396 | Shigella sonnei |
| E1720 | NZ_CP006639 (PCN061p3) | NZ_CP006639.1 | 100 | 2 | 0.00032144 | Escherichia coli |
| | NZ_CP018439 (pKp_Goe_917-8) | NZ_CP018439.1 | 100 | 2 | 0.001034661 | Klebsiella pneumoniae |
| | NZ_CP011143 (pVR50I) | NZ_CP011143.1 | 98.7757732 | 1 | 0.00064433 | Escherichia coli |
| E1721 | NZ_CP011141 (pVR50G) | NZ_CP011141.1 | 100 | 2 | 0.000957396 | Escherichia coli |

**Supplementary Table 5: The table shows the insertion sequences in each sample with IS group, coverage, reads count, and IS copy number.**

| Sample | IS | IS Group | Coverage | Reads Count | IS Copy Number |
|---|---|---|---|---|---|
| E1701 | IS609 | IS200/IS605 | 90.84668192 | 87 | 0.049771167 |
| | ISEc12 | IS21 | 100 | 101 | 0.039132119 |
| | IS26 | IS6 | 100 | 174 | 0.212195122 |
| | ISCfr1 | IS1182 | 97.95918367 | 50 | 0.030921459 |
| | IS6100 | IS6 | 100 | 37 | 0.042045455 |
| E1702 | IS26 | IS6 | 100 | 214 | 0.26097561 |
| | ISKox3 | ISL3 | 100 | 126 | 0.095744681 |
| | ISEc12 | IS21 | 100 | 62 | 0.024021697 |
| | IS1R | IS1 | 85.9375 | 11 | 0.014322917 |
| | IS6100 | IS6 | 91.81818182 | 13 | 0.014772727 |
| E1703 | IS26 | IS6 | 100 | 172 | 0.209756098 |
| | ISKox3 | ISL3 | 100 | 136 | 0.103343465 |
| | ISEc12 | IS21 | 89.84889578 | 37 | 0.014335529 |
| | IS1X2 | IS1 | 93.88020833 | 9 | 0.01171875 |
| | MITEEc1 | IS630 | 100 | 1 | 0.008130081 |
| E1704 | ISEc12 | IS21 | 98.41146842 | 52 | 0.02014723 |
| | IS26 | IS6 | 100 | 56 | 0.068292683 |
| | ISCfr1 | IS1182 | 94.31045145 | 17 | 0.010513296 |
| | IS1397 | IS3 | 86.31284916 | 8 | 0.005586592 |
| | IS6100 | IS6 | 100 | 11 | 0.0125 |
| E1705 | IS26 | IS6 | 100 | 167 | 0.203658537 |
| | MITEEc1 | IS630 | 87.80487805 | 1 | 0.008130081 |
| | IS1X2 | IS1 | 98.046875 | 15 | 0.01953125 |
| | IS6100 | IS6 | 100 | 45 | 0.051136364 |
| | IS2 | IS3 | 99.69947408 | 28 | 0.021036814 |
| | ISEc12 | IS21 | 97.52034095 | 47 | 0.018209996 |
| E1706 | ISCfr1 | IS1182 | 100 | 42 | 0.025974026 |
| | IS6100 | IS6 | 93.18181818 | 14 | 0.015909091 |
| | IS26 | IS6 | 100 | 99 | 0.120731707 |
| | MITEEc1 | IS630 | 100 | 3 | 0.024390244 |
| | IS2 | IS3 | 85.80015026 | 18 | 0.013523666 |
| E1707 | ISEc12 | IS21 | 100 | 145 | 0.056179775 |
| | ISCfr1 | IS1182 | 100 | 46 | 0.028447743 |
| | IS26 | IS6 | 100 | 251 | 0.306097561 |
| | IS6100 | IS6 | 100 | 32 | 0.036363636 |
| | IS200C | IS200/IS605 | 100 | 28 | 0.039492243 |
| E1708 | ISEcp1 | IS1380 | 100 | 336 | 0.202898551 |
| | ISCro1 | IS66 | 100 | 468 | 0.173397555 |
| | ISEc23 | IS66 | 100 | 111 | 0.043838863 |
| | IS6100 | IS6 | 100 | 37 | 0.042045455 |
| | ISEc76 | IS110 | 100 | 81 | 0.059210526 |
| | ISEc12 | IS21 | 100 | 122 | 0.047268501 |
| | IS26 | IS6 | 100 | 147 | 0.179268293 |
| | IS421 | IS4 | 93.6661699 | 28 | 0.020864382 |
| E1709 | ISCfr1 | IS1182 | 100 | 76 | 0.047000618 |
| | IS26 | IS6 | 100 | 335 | 0.408536585 |
| | ISEc12 | IS21 | 100 | 107 | 0.0414568 |
| | IS200C | IS200/IS605 | 100 | 23 | 0.032440056 |
| | IS609 | IS200/IS605 | 87.58581236 | 89 | 0.050915332 |
| | IS6100 | IS6 | 100 | 24 | 0.027272727 |
| | MITEEc1 | IS630 | 99.18699187 | 1 | 0.008130081 |

| | | | | | |
|---|---|---|---|---|---|
| E1710 | IS26 | IS6 | 100 | 294 | 0.358536585 |
| | ISEc12 | IS21 | 100 | 147 | 0.056954669 |
| | ISCfr1 | IS1182 | 100 | 79 | 0.048855906 |
| | IS6100 | IS6 | 100 | 29 | 0.032954545 |
| | IS200C | IS200/IS605 | 100 | 21 | 0.029619182 |
| | MITEEc1 | IS630 | 100 | 2 | 0.016260163 |
| E1711 | IS200C | IS200/IS605 | 100 | 12 | 0.016925247 |
| | ISEc12 | IS21 | 100 | 163 | 0.063153816 |
| | IS26 | IS6 | 100 | 388 | 0.473170732 |
| | ISCfr1 | IS1182 | 100 | 85 | 0.052566481 |
| | IS6100 | IS6 | 100 | 46 | 0.052272727 |
| E1712 | IS26 | IS6 | 100 | 98 | 0.119512195 |
| | ISCfr1 | IS1182 | 86.45640074 | 15 | 0.009276438 |
| | ISEc23 | IS66 | 100 | 56 | 0.022116904 |
| | IS2 | IS3 | 96.69421488 | 15 | 0.011269722 |
| | IS1R | IS1 | 89.0625 | 15 | 0.01953125 |
| E1713 | IS26 | IS6 | 100 | 289 | 0.352439024 |
| | IS6100 | IS6 | 100 | 38 | 0.043181818 |
| | ISEc12 | IS21 | 100 | 227 | 0.087950407 |
| | IS1F | IS1 | 97.78645833 | 7 | 0.009114583 |
| | ISVsa5 | IS4 | 93.67945824 | 36 | 0.027088036 |
| | IS150 | IS3 | 99.58419958 | 33 | 0.022869023 |
| | IS1X2 | IS1 | 85.02604167 | 16 | 0.020833333 |
| | IS200C | IS200/IS605 | 100 | 15 | 0.021156559 |
| E1714 | IS26 | IS6 | 100 | 344 | 0.419512195 |
| | ISVsa3 | IS91 | 100 | 15 | 0.015353122 |
| | IS150 | IS3 | 99.0990991 | 107 | 0.074151074 |
| | IS6100 | IS6 | 100 | 64 | 0.072727273 |
| | IS2 | IS3 | 86.47633358 | 26 | 0.019534185 |
| | ISEc12 | IS21 | 85.89693917 | 15 | 0.005811701 |
| | ISEc17 | IS3 | 98.88712242 | 25 | 0.019872814 |
| | IS1R | IS1 | 86.45833333 | 36 | 0.046875 |
| E1715 | IS26 | IS6 | 100 | 702 | 0.856097561 |
| | IS6100 | IS6 | 100 | 90 | 0.102272727 |
| | ISEc12 | IS21 | 100 | 302 | 0.117008911 |
| | IS200C | IS200/IS605 | 100 | 36 | 0.05077574 |
| E1716 | ISAba125 | IS30 | 100 | 111 | 0.102115915 |
| | IS26 | IS6 | 100 | 160 | 0.195121951 |
| | ISVsa5 | IS4 | 92.85176825 | 23 | 0.017306245 |
| | ISEc12 | IS21 | 100 | 79 | 0.030608291 |
| | ISKox3 | ISL3 | 100 | 101 | 0.07674772 |
| | IS150 | IS3 | 96.18849619 | 36 | 0.024948025 |
| | IS5D | IS5 | 100 | 64 | 0.049883087 |
| | MITEEc1 | IS630 | 100 | 1 | 0.008130081 |
| | IS15DIV | IS6 | 85.12195122 | 22 | 0.026829268 |
| | IS6100 | IS6 | 99.65909091 | 20 | 0.022727273 |
| | ISVsa3 | IS91 | 87.10337769 | 12 | 0.012282497 |

| | | | | | |
|---|---|---|---|---|---|
| E1717 | IS26 | IS6 | 100 | 437 | 0.532926829 |
| | IS1294 | IS91 | 100 | 83 | 0.049170616 |
| | ISAba125 | IS30 | 100 | 145 | 0.133394664 |
| | ISKox3 | ISL3 | 100 | 163 | 0.123860182 |
| | IS5D | IS5 | 100 | 124 | 0.09664848 |
| | ISKpn19 | ISKra4 | 100 | 68 | 0.02385128 |
| | ISEc12 | IS21 | 100 | 220 | 0.08523828 |
| | IS5075 | IS110 | 98.19140919 | 45 | 0.033911078 |
| | ISEcp1 | IS1380 | 100 | 73 | 0.044082126 |
| | IS150 | IS3 | 87.04088704 | 21 | 0.014553015 |
| | IS4321R | IS110 | 100 | 31 | 0.023378582 |
| | IS1X4 | IS1 | 100 | 30 | 0.0390625 |
| | MITEEc1 | IS630 | 100 | 1 | 0.008130081 |
| | IS6100 | IS6 | 100 | 55 | 0.0625 |
| E1718 | ISEcp1 | IS1380 | 93.65942029 | 38 | 0.02294686 |
| | ISEc12 | IS21 | 100 | 126 | 0.048818287 |
| | IS26 | IS6 | 100 | 397 | 0.484146341 |
| | IS1294 | IS91 | 100 | 56 | 0.033175355 |
| | ISKox3 | ISL3 | 100 | 103 | 0.078267477 |
| | ISKpn19 | ISKra4 | 100 | 171 | 0.059978955 |
| | IS150 | IS3 | 87.8031878 | 11 | 0.007623008 |
| | IS6100 | IS6 | 100 | 65 | 0.073863636 |
| | ISAba125 | IS30 | 100 | 89 | 0.081876725 |
| | IS1X4 | IS1 | 100 | 24 | 0.03125 |
| | IS5075 | IS110 | 98.49284099 | 33 | 0.024868124 |
| | IS5D | IS5 | 100 | 66 | 0.051441933 |
| | IS4321R | IS110 | 93.36349925 | 17 | 0.012820513 |
| E1719 | IS1294 | IS91 | 100 | 42 | 0.024881517 |
| | ISEc12 | IS21 | 100 | 105 | 0.040681906 |
| | ISKpn19 | ISKra4 | 100 | 105 | 0.036829183 |
| | ISKox3 | ISL3 | 100 | 122 | 0.092705167 |
| | IS26 | IS6 | 100 | 395 | 0.481707317 |
| | ISAba125 | IS30 | 100 | 130 | 0.119595216 |
| | IS4321R | IS110 | 100 | 26 | 0.019607843 |
| | IS5D | IS5 | 97.19407638 | 75 | 0.058456742 |
| | ISEcp1 | IS1380 | 88.58695652 | 39 | 0.023550725 |
| | IS5075 | IS110 | 100 | 28 | 0.021100226 |
| | IS1X4 | IS1 | 98.828125 | 19 | 0.024739583 |
| | IS6100 | IS6 | 100 | 56 | 0.063636364 |
| | IS150 | IS3 | 89.11988912 | 14 | 0.00970201 |
| | MITEEc1 | IS630 | 100 | 2 | 0.016260163 |
| E1720 | ISPa61 | ISL3 | 87.77686628 | 74 | 0.060705496 |
| | ISPst6 | IS110 | 100 | 49 | 0.035740336 |
| | ISKpn19 | ISKra4 | 95.19466854 | 36 | 0.012627148 |
| | ISKox3 | ISL3 | 100 | 26 | 0.019756839 |
| | ISPst3 | IS21 | 85.37989256 | 33 | 0.012663085 |
| | IS26 | IS6 | 100 | 45 | 0.054878049 |
| | IS4321R | IS110 | 100 | 20 | 0.015082956 |
| | ISAchd1 | IS1595 | 92.69717624 | 12 | 0.011684518 |
| E1721 | ISEc12 | IS21 | 100 | 45 | 0.017435103 |
| | IS26 | IS6 | 100 | 199 | 0.242682927 |
| | IS903B | IS5 | 100 | 58 | 0.05487228 |
| | IS2 | IS3 | 91.81066867 | 20 | 0.015026296 |
